# Cmr1/WDR76 defines a nuclear genotoxic stress body linking genome integrity and protein quality control

**DOI:** 10.1038/ncomms7533

**Published:** 2015-03-30

**Authors:** Irene Gallina, Camilla Colding, Peter Henriksen, Petra Beli, Kyosuke Nakamura, Judith Offman, David P. Mathiasen, Sonia Silva, Eva Hoffmann, Anja Groth, Chunaram Choudhary, Michael Lisby

**Affiliations:** 1 Department of Biology, University of Copenhagen, Room 4.1.07, Copenhagen N DK-2200, Denmark; 2 The Novo Nordisk Foundation Center for Protein Research, Faculty of Health and Medical Sciences, University of Copenhagen, Copenhagen N DK-2200, Denmark; 3 Biotech Research and Innovation Centre (BRIC) and Centre for Epigenetics, University of Copenhagen, Copenhagen N DK-2200, Denmark; 4 MRC, Centre for Genome Damage and Stability, School of Life Sciences, University of Sussex, Brighton BN1 9RH, UK

## Abstract

DNA replication stress is a source of genomic instability. Here we identify changed mutation rate 1 (Cmr1) as a factor involved in the response to DNA replication stress in *Saccharomyces cerevisiae* and show that Cmr1—together with Mrc1/Claspin, Pph3, the chaperonin containing TCP1 (CCT) and 25 other proteins—define a novel intranuclear quality control compartment (INQ) that sequesters misfolded, ubiquitylated and sumoylated proteins in response to genotoxic stress. The diversity of proteins that localize to INQ indicates that other biological processes such as cell cycle progression, chromatin and mitotic spindle organization may also be regulated through INQ. Similar to Cmr1, its human orthologue WDR76 responds to proteasome inhibition and DNA damage by relocalizing to nuclear foci and physically associating with CCT, suggesting an evolutionarily conserved biological function. We propose that Cmr1/WDR76 plays a role in the recovery from genotoxic stress through regulation of the turnover of sumoylated and phosphorylated proteins.

## Abstract (web summary)

Defects in the DNA replication checkpoint can lead to genomic instability and cancer. Here the authors show that Cmr1/WDR76 participates in the DNA replication stress response and—along with several other components—defines a new cellular compartment that forms during cellular stress.

## Graphical abstract

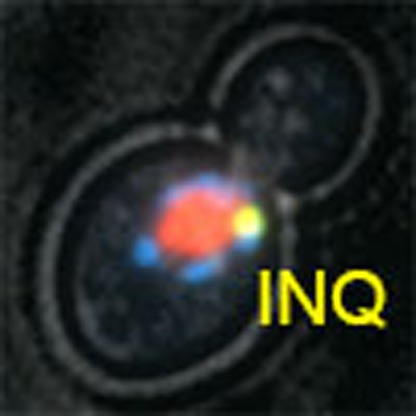

Faithful completion of DNA replication is essential for cell survival and for inheritance of the genetic information. Replication fork stalling at DNA lesions leads to activation of the replication checkpoint, which in *S. cerevisiae* relies on the recruitment of the checkpoint kinase Mec1/ATR to RPA (replication protein A)-coated single-stranded DNA, arising from the uncoupling of the polymerase and the mini-chromosome maintenance (MCM) helicase^1^. Mec1-dependent phosphorylation of the checkpoint mediator Mrc1/Claspin leads to the recruitment and activation of the effector kinase Rad53 (refs 2, 3). The replication checkpoint induces posttranslational modification of the clamp loader PCNA (proliferating cell nuclear antigen), promoting the repair or bypass of the lesion^4^. Failure to activate the replication checkpoint leads to severe chromosomal instability, a major trigger for cancer in humans^5^.

Resumption of DNA replication after checkpoint activation relies both on the repair or bypass of the lesion and on the inactivation of checkpoint signalling. The latter requires dephosphorylation of Rad53 by the PP4 phosphatase Pph3-Psy2-Psy4 (ref. 6) and proteasome-dependent degradation of fork-associated factors such as Mrc1 (ref. 7). Specifically, Mrc1 has recently been identified as a target of the ubiquitin ligase complex SCF–Dia2 (ref. 7). Dia2 directly binds Mrc1 and promotes its ubiquitylation and proteasomal degradation in response to replication stress. This recovery pathway appears to act in parallel with dephosphorylation of Rad53, as *DIA2* and *PPH3* show negative genetic interaction in the presence of replication stress^8^.

Cmr1 (changed mutation rate 1) is a nuclear WD40 protein of unknown function^9^,^10^, which has recently appeared in several large-scale studies. First, Cmr1 was described as a histone-related protein^11^, with DNA-binding capacity *in vitro* and with the ability to accumulate on chromatin in response to ultraviolet irradiation^12^. Furthermore, in a genome-wide screen Cmr1 was found to specifically respond to methyl methanesulfonate (MMS)-induced damage, relocalizing to nuclear foci of undetermined nature^13^. Finally, *in silico* clustering analyses suggest that *CMR1* is co-expressed with genes involved in processes related to DNA metabolism^14^. Taken together, these data suggest a role for Cmr1 in genome maintenance. Here we identify Cmr1 in two independent screens and provide the first extensive functional characterization of Cmr1 and the nuclear structure that it forms in response to replication stress and proteasome inhibition. Together with the replication checkpoint proteins Mrc1, Pph3 and 25 other proteins, Cmr1 defines a novel intranuclear quality control compartment (INQ) for the sequestering of phosphorylated, sumoylated and ubiquitylated proteins. Our findings document a novel connection between the cellular response to DNA replication stress and turnover of replication stress factors.

## Results

### Identification of Cmr1 as a genome maintenance factor

**Figure 1:**
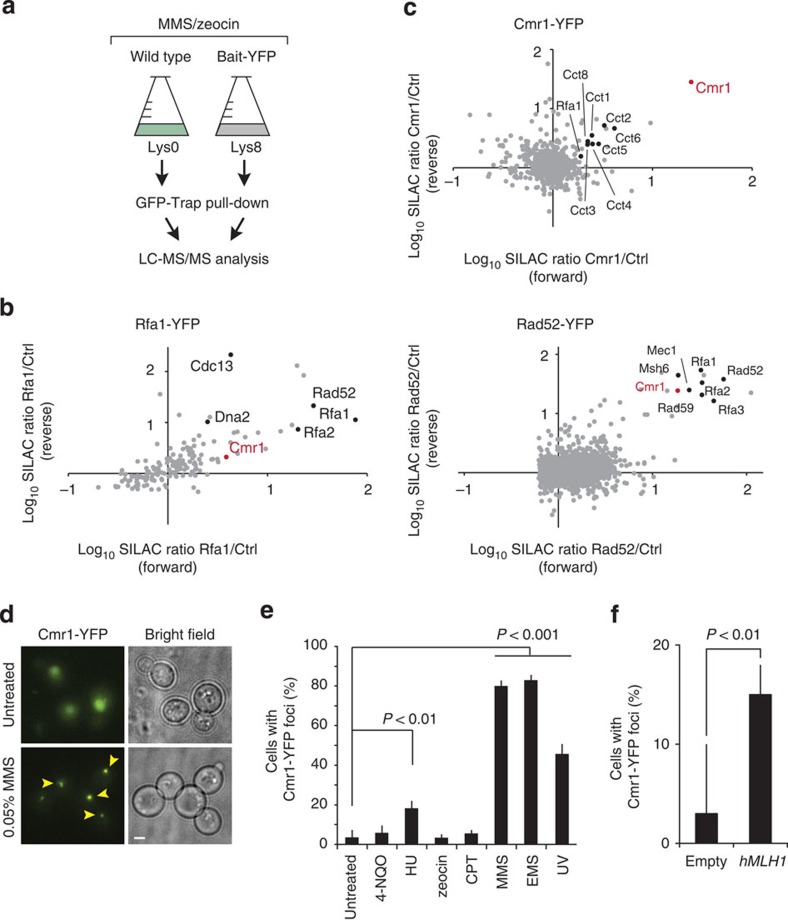
Cmr1 associates with RPA-bound chromatin. (**a**) Representation of the workflow used for the SILAC-based identification of protein complexes associated with DNA repair factors. Yeast strains expressing YFP-tagged (IG54-11D and IG46-1B) or untagged (IG45-8A) proteins were cultured in SILAC media and harvested in log phase after treatment with DNA-damaging agents. Protein complexes from SILAC lysates were affinity purified separately with GFP-Trap. Proteins were trypsin proteolysed and peptides were identified by liquid chromatography (LC)–MS/MS. (**b**) Identification of Rfa1-YFP and Rad52-YFP interacting proteins. The plots show log(10) SILAC ratios from GFP-tagged bait versus control from forward and reverse (SILAC label swap) experiments. Dots indicate identified proteins. Cmr1 is highlighted in red and some of the known interactors for Rfa1 and Rad52 are indicated in black in the respective plots. (**c**) Identification of Cmr1 interacting proteins. Cmr1-YFP (IG71-2B) was used as bait for the pull down; Cmr1 is highlighted in red; CCT-chaperonin complex subunits and Rfa1 are indicated in black. (**d**) Cmr1 relocalization into foci. Representative images of untreated and MMS-treated cells are shown. Arrowheads indicate selected foci. Scale bar, 2 μm. (**e**) Quantification of Cmr1 foci. Cmr1-YFP localization was examined by fluorescence microscopy in IG66. Cells were grown to exponential phase and imaged after treatment with zeocin (200 μg ml^−1^), MMS (0.05%), CPT (5 μg ml^−1^), 4-NQO (0.2 μg ml^−1^), HU (200 mM), EMS (0.5%) for 2 h, or 1 h after ultraviolet irradiation (25 J m^−2^). Error bars represent 95% confidence intervals. Two to 3 replicates of 100–200 cells were analysed for each condition. (**f**) hMLH1 expression causes accumulation of Cmr1 foci. Cells expressing Cmr1-YFP (IG66) were transformed with pEH333 for ectopic expression of hMLH1 or with an empty vector (pEH334). Error bars represent 95% confidence intervals. Two to 3 replicates of 100–200 cells were analysed for each strain.

In an effort to identify new factors involved in the maintenance of genome stability, a series of stable isotope labelling by amino acids in cell culture (SILAC)-based mass spectrometry (MS) experiments were performed under conditions wherein the replication protein Rfa1 and the recombination protein Rad52 were induced to relocalize to DNA repair foci by DNA damage before protein extraction and pull down using a yellow fluorescent protein (YFP) tag (Fig. 1a). This approach identified a collection of proteins, including the WD40-domain protein Cmr1 (Fig. 1b). Further, the physical association between Cmr1 and the RPA complex, which has been reported in several independent large-scale studies^11^,^15^,^16^, was confirmed by reverse pull down using Cmr1-YFP as the bait (Fig. 1c and Supplementary Data 1). In an independent systematic genome-wide screen for mutants that change mutation rates, we found that *cmr1*Δ suppressed the otherwise elevated mutation rates resulting from expression of the human mismatch repair (MMR) gene *MLH1* (*hMLH1*) in *S. cerevisiae* (Supplementary Fig. 1)^17^. Further analyses to assess the involvement of Cmr1 in MMR showed that deletion of *CMR1* increases frameshift mutation rates in an *MRC1*- and *MLH1*-dependent manner, but additively increases the overall *CAN1* forward mutation rate in conjunction with *msh2*Δ and *mlh1*Δ (Table 1a,b). These findings indicate a defect in replication rather than in MMR *per se*.

### Cell cycle-independent formation of perinuclear Cmr1 foci

The observation that endogenously tagged Cmr1 relocalized from diffusely nuclear to a distinct focus on hMLH1 expression in yeast or after treatment with MMS, ultraviolet irradiation or hydroxyurea (HU;Fig. 1d–f), supported our hypothesis of Cmr1 being recruited to a replication or DNA repair factory. Surprisingly, but consistent with a recent report^13^, the Cmr1 focus did not co-localize with any known nuclear structures such as telomeres (Cdc13), nuclear pore complex (Nup49), MMR (Pms1), spindle pole body (Spc110), recombination (Rad52), replication (Pol30 and Rfa1), or the nucleolus (Nop1, data not shown). We therefore concluded that Cmr1 defines a novel nuclear compartment that forms in response to genotoxic stress.

**Figure 2:**
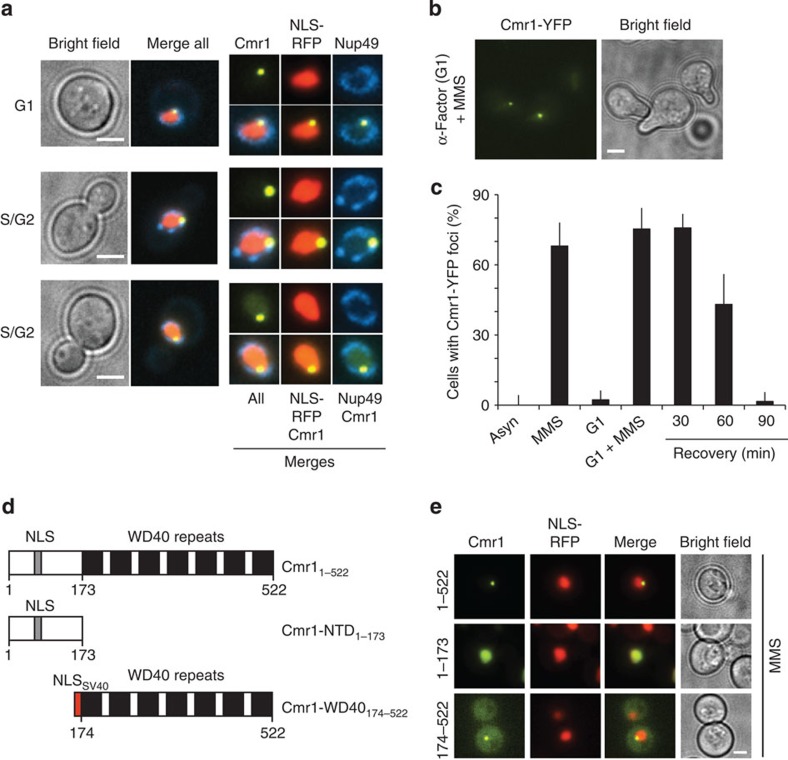
Cmr1 localizes into perinuclear foci. (**a**) Cmr1 foci localize at the nuclear periphery. Cells expressing Cmr1-YFP (IG66) were transformed with pNEB21 for expression of Nup49-CFP and pML84 for expression of NLS-yEmRFP, and treated with 0.05% MMS. Cmr1 localization relative to the nucleoplasm and the nuclear membrane is shown in both unbudded (G1) and budded (S/G2) cells. Nuclear zooms are at 150%. (**b**) Cmr1 foci in α-factor-arrested cells. Cells (IG66) were cultured with α-factor for 1 h, followed by treatment with 0.05% MMS for 2 h. (**c**) Quantification of Cmr1 foci. The percentage of cells (IG66) with Cmr1-YFP foci was quantified in asynchronous and G1-arrested populations, before and after treatment with 0.05% MMS for 1 h, and 30, 60 and 90 min after the drug was removed. Two to 3 replicates of 100–200 cells were analysed for each condition. Error bars represent 95% confidence intervals. (**d**) Schematic representation of Cmr1 and truncation constructs. (**e**) The WD40 domain of Cmr1 is necessary and sufficient for relocalization into foci. Cmr1-YFP constructs containing the full-length protein (pIG13), the N-terminal (pIG14) or WD40-containing portion of the protein (pIG15) were expressed from a 2-μm plasmid in cells (ML657) harbouring NLS-yEmRFP, and relocalization of Cmr1 into nuclear foci was monitored after treatment with 0.05% MMS. Scale bars, 2 μm.

Given the vicinity of Cmr1 foci to the nuclear periphery, we further examined Cmr1 foci relative to the nuclear membrane in asynchronously growing and G1-arrested cells. Cmr1 foci formed with similar efficiency in G1 and S/G2 cells, consistently localized internally to the nuclear envelope (Nup49-CFP), and disassembled within 90 min after ultraviolet irradiation or on removal of MMS (Fig. 2a–c and Supplementary Fig. 2a,b). Accumulation into perinuclear foci was also observed for the endogenous Cmr1 (Supplementary Fig. 2c,d), indicating that its localization is not an artefact of the YFP tagging. Structurally, Cmr1 is predicted to consist of a carboxy-terminal WD40 domain and of an amino-terminal unstructured region. Using plasmids expressing YFP fusions of the N-terminal domain (NTD) or the WD40 domain, we found that the WD40 domain was necessary and sufficient for the re-localization of Cmr1 into foci (Fig. 2d,e).

### Cmr1 marks an intranuclear quality control compartment

**Figure 3:**
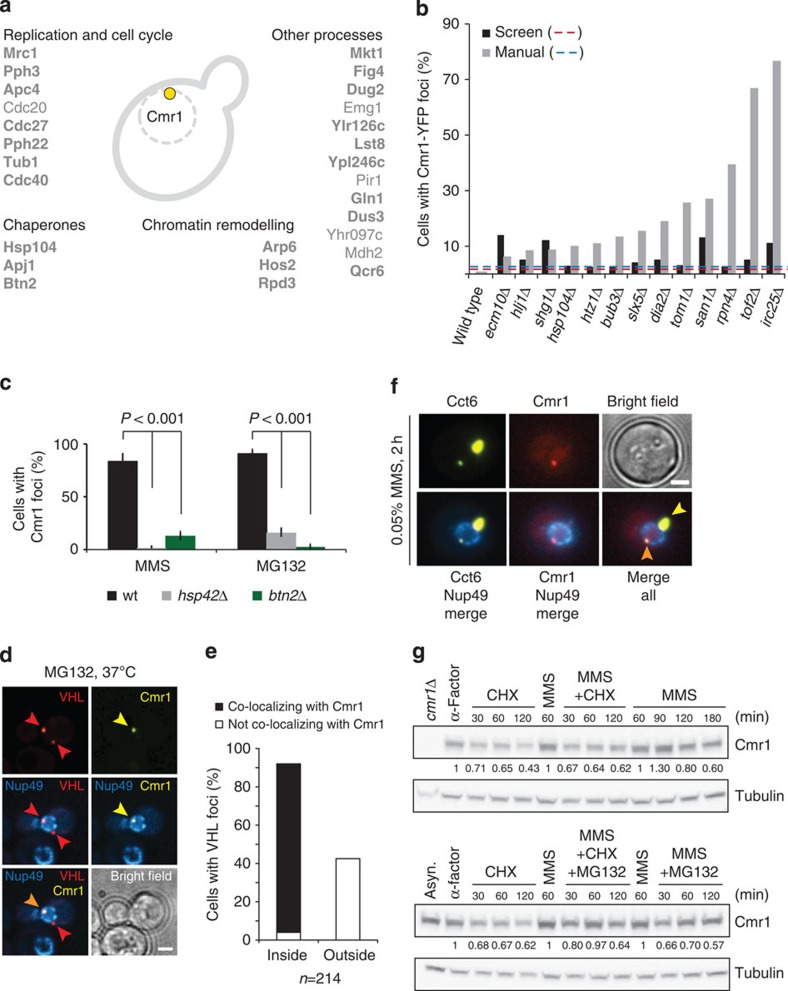
Characterization of INQ. (**a**) Genome-wide analysis of Cmr1 co-localizing proteins. Haploid cells expressing GFP-tagged query proteins and Rad52-RFP as a nuclear marker (IG72-5C) were imaged by high-content fluorescence microscopy, untreated or treated for 2 h with 75 μg ml^−1^ MG132. Proteins re-localizing into perinuclear foci were further tested for co-localization with Cmr1. Confirmed co-localizing proteins are listed. Proteins that also co-localize with Cmr1 after MMS treatment are highlighted in bold. (**b**) Cmr1 foci are induced by genetic impairment of proteasome function. Gene deletion strains expressing Cmr1-YFP and NLS-yEmRFP were imaged by high-content fluorescence microscopy. Strains exhibiting more than threefold increase in the percentage of spontaneous Cmr1 foci compared with wild type were manually retested. Only the mutants giving a result significantly different from the wild type are reported in the figure. Six mutants (*arp6Δ*, *slx1Δ*, *fpr1Δ*, *whi2Δ*, *sgs1Δ* and *csm2Δ*) exhibited elevated Cmr1-YFP foci levels in the screen, but not on manual retesting. Red and blue dashed lines represent the threefold thresholds for the automated and manual analyses, respectively. IG66 served as the wild-type reference strain for the manual re-testing. (**c**) Cmr1 foci are dependent on Hsp42 and Btn2. Cells deleted for *BTN2* (IG239-2B) or *HSP42* (IG238-9D) and expressing Cmr1-YFP were treated with MMS or MG132 for 2 h. Two to 3 replicates of 100–200 cells were analysed for each condition. Error bars represent 95% confidence intervals. (**d**) Cmr1 defines INQ. Cells expressing Cmr1-YFP (IG66), Cherry-VHL (pESC-mCherry-VHL) and Nup49-CFP (pNEB21) were grown at 25 °C to log phase in synthetic complete medium lacking tryptophan and uracil, and with 2% raffinose as a carbon source. Cherry-VHL expression was induced by addition of 3% galactose for 3 h, followed by a shift to 37 °C and treatment with 75 μg ml^−1^ MG132 in 2% glucose for 1 h before imaging. Arrowheads mark VHL and Cmr1 foci. Images were deconvolved using the Volocity software (PerkinElmer). Scale bar, 2 μm. (**e**) Quantification of the foci described in **d**. Cherry-VHL foci (*n*=214) located inside and outside the nuclear periphery were assessed for co-localization with Cmr1-YFP. (**f**) Cmr1 and the CCT–chaperonin complex co-localize at perinuclear foci. Cells express Cmr1-yEmRFP (IG111), Nup49-CFP (pNEB21) and Cct6-YFP (pIG20). Orange arrowhead, Cmr1 and Cct6 co-localizing at a perinuclear focus. Yellow arrowhead, Cct6 focus. Scale bar, 2 μm. (**g**) Cmr1 is not degraded during the DNA-damage response. G1-arrested cells (IG174) were released into YPD containing 200 μg ml^−1^ cycloheximide (CHX) or 0.05% MMS. After 60 min of MMS treatment, CHX and 75 μg ml^−1^ MG132 or CHX and MG132 were added. Cmr1-TAP and tubulin were analysed by immunoblotting, using *cmr1*Δ (DP1) as a negative control. Cmr1 protein levels relative to the sample taken before addition of CHX are indicated below the blot.

To gain insight into the biological processes represented by Cmr1 foci, we screened a collection of 4800 green fluorescent protein (GFP)-tagged proteins for co-localization with Cmr1 (ref. 10). We took advantage of the observation that Cmr1 perinuclear foci were also induced by proteasome inhibition (MG132; Supplementary Fig. 2e), to avoid induction of DNA repair foci^13^. Strains exhibiting MG132-induced perinuclear foci were individually re-tested for co-localization with Cmr1-yEmRFP, yielding a list of 27 proteins that form Cmr1-co-localizing foci in response to MG132 (Fig. 3a). Eighty-one per cent (22/27) of these proteins also co-localized with Cmr1 after MMS treatment. Notably, proteins implicated in chromosome organization, mitotic cell cycle, spindle organization and dephosphorylation were overrepresented among these proteins (Supplementary Fig. 3a–c). The hits included regulators of the S-phase checkpoint response (Mrc1 and Pph3) and components of the anaphase-promoting complex (Cdc20, Cdc27 and Apc4), chaperones (Hsp104 and Apj1) and histone deacetylases (Hos2 and Rpd3)^13^.

To further characterize the properties of Cmr1 foci, we screened the collection of 5,200 non-essential gene deletion mutants^18^ to determine the genetic requirements for the formation of Cmr1 foci in the presence of replication stress, and to identify gene deletions that would lead to spontaneous accumulation of Cmr1 foci. Consistent with their induction by proteasome inhibition, the top-scoring hits for increased spontaneous Cmr1 foci were mutants involved in proteasomal degradation of nuclear targets (*irc25*Δ, *rpn4*Δ, *san1*Δ, *tom1*Δ and *dia2*Δ) and ubiquitylation of sumoylated proteins (*slx5*Δ; Fig. 3b). This suggests that even in the absence of acute replication stress, Cmr1 is channelled towards a perinuclear compartment as part of an ubiquitin-dependent degradation pathway and points to Cmr1 foci as nuclear sites for protein degradation. To test this hypothesis further, we monitored the enrichment of the proteasome subunit Rpn11 at Cmr1 foci. After MG132 treatment, Rpn11 was observed at 13% (11/81) of the Cmr1 foci, indicating that the proteasome has the potential to target proteins for degradation at the perinuclear structure defined by Cmr1 (Supplementary Fig. 3d).

The only non-essential genes required for Cmr1 focus formation were *HSP42* and *BTN2* (Fig. 3c). Hsp42 is a small heat shock protein with chaperone activity, which has recently been found to be essential for organization and sorting of protein aggregates into deposition sites in yeast^19^,^20^. Similarly, Btn2 has been identified in a recent study as a crucial regulator of the cellular protein quality control^21^. Interestingly, both Hsp42 and Btn2 have been implicated in the partitioning of misfolded proteins between two recently identified protein quality compartments, the juxtanuclear quality control (JUNQ) and the cytosolic insoluble protein deposit (IPOD)^21^,^22^,^23^.

To assess whether Cmr1 foci coincide with JUNQ, the localization of the unassembled von Hippel–Lindau (VHL) tumour suppressor VHL-GFP^24^ and Cmr1-yEmRFP was monitored under conditions leading to VHL misfolding and accumulation into deposition sites^22^. Indeed, Cmr1 co-localized with VHL at the nuclear periphery in >50% of the cells, but was never observed at the perivacuolar IPOD (Supplementary Fig. 4a). As Cmr1 foci resemble JUNQ but are strictly nuclear, we name this structure intranuclear quality control (INQ). In agreement with our observation that INQ is a nuclear structure, we observed that >95% of the nuclear mCherry-VHL foci co-localized with Cmr1, while none of the juxtanuclear or peripheral cytoplasmic VHL foci co-localized with Cmr1 (Fig. 3d,e), indicating that INQ is a nuclear variant of JUNQ that can be distinguished by the presence of Cmr1. Notably, MG132-induced nuclear VHL foci only required *BTN2*, while MMS-induced VHL foci also required *HSP42*, indicating that the sorting of Cmr1 and VHL to INQ could occur by different mechanisms (Supplementary Fig. 4b,c).

The physical interaction of Cmr1 with all eight subunits of the chaperonin containing TCP1 (CCT) (Fig. 1c) prompted us to examine the recruitment of CCT to INQ. On expression of Cct6-YFP, ~60% of the cells exhibited one to three cytoplasmic and nuclear Cct6 foci, typically one very bright focus in the cytoplasm and one weaker focus at the nuclear periphery (Fig. 3f). Importantly, ~12% of the Cct6-YFP perinuclear foci co-localized with Cmr1, while no co-localization was observed with the brighter cytoplasmic focus. Notably, Cmr1 itself did not behave similar to a misfolded protein, as its relocalization to INQ was not perturbed by the actin or microtubule depolymerizing drugs latrunculin B and nocodazole, respectively, a requirement previously demonstrated for relocalization of misfolded proteins to JUNQ (Supplementary Fig. 4d,e and data not shown)^20^,^22^. Moreover, the stability of Cmr1 did not change significantly after MMS treatment (Fig. 3g), suggesting that Cmr1 could be a mediator rather than a target of proteasomal degradation. Taken together, Cmr1 defines a novel intranuclear protein quality control structure, INQ, for proteasome-dependent turnover and/or refolding of proteins primarily involved in DNA metabolism and cell cycle control.

### Cmr1 interacts with chromatin and replication factors

**Figure 4:**
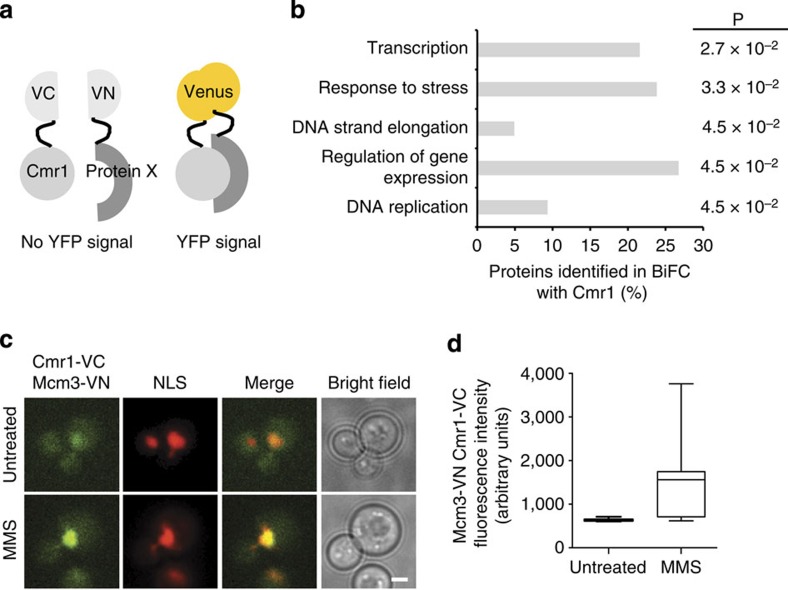
Cmr1 interacts with chromatin and replication factors. (**a**) Schematic representation of the principle of the bimolecular fluorescence complementation (BiFC) assay. N-terminal (VN) and C-terminal (VC) non-fluorescent fragments of Venus fluorescent protein are fused to putative interacting proteins, to assess their physical association by the appearance of a fluorescence signal. (**b**) Gene Onthology (GO) enrichment analysis of Cmr1 interaction partners in BiFC. Significantly overrepresented GO biological process terms are shown. Bars indicate the percentage of Cmr1 interactors belonging to the indicated GO term as determined using BinGO^69^. *P*-values were calculated by Fisher’s *t*-test and corrected using the Benjamini and Hochberg false discovery rate correction. (**c**) Cmr1 interaction with Mcm3 is enhanced by MMS. The strain from the BiFC screen expressing Cmr1-VC, Mcm3-VN and NLS-yEmRFP was subjected to fluorescence microscopy before and after treatment with 0.05% MMS for 2 h. Scale bar, 2 μm. (**d**) Quantification of the intensity of the Cmr1-Mcm3 interaction signal in cells from experiment in **c**. Two to 3 replicates of 100–200 cells were analysed for each condition. The box plot displays nuclear fluorescence intensities in arbitrary units (AU), where the line across the box identifies the median sample value, the ends of the box are the 25th and 75th percentiles, and whiskers represent minimum and maximum values.

As Cmr1 relocalization was not coupled to its degradation, we reasoned that Cmr1 might facilitate another aspect of INQ function. To identify possible targets of Cmr1 function, we performed a systematic genome-wide screen for *in situ* physical interactions using bimolecular fluorescence complementation^10^ (Fig. 4a). A query strain expressing Cmr1 fused to the C-terminal fragment of Venus (Cmr1-VC) was crossed to a library of 5,809 strains expressing proteins fused to the Venus N terminus^25^ (VN) and physical interactions (VN–VC) were assessed by the appearance of a Venus fluorescence signal. We found 79 proteins to be unique interactors of Cmr1 compared with other bait proteins tested (Supplementary Table 1 and unpublished data). These showed a significant overrepresentation of Gene Onthology biological process terms related to DNA replication, transcription and regulation of gene expression (Fig. 4b), consistent with the notion that Cmr1 is a component of chromatin^11^,^12^. However, none of the other INQ factors were found to interact with Cmr1. The VN–VC interaction signals were mainly nuclear and a subnuclear localization pattern into foci or speckles was often observed (Supplementary Fig. 5). Interestingly, four replication fork proteins, Mcm3, Pri1, Rfc2 and Rfc3, were identified in this screen (Fig. 4c,d and Supplementary Fig. 5). Notably, the Mcm3-Cmr1 interaction significantly increased by about twofold in response to MMS treatment (Fig. 4d). Taken together, this analysis confirms that Cmr1 interactions are enriched for chromatin-associated factors^11^,^12^.

### Btn2 promotes Mrc1 turnover and relocalization to INQ

**Figure 5:**
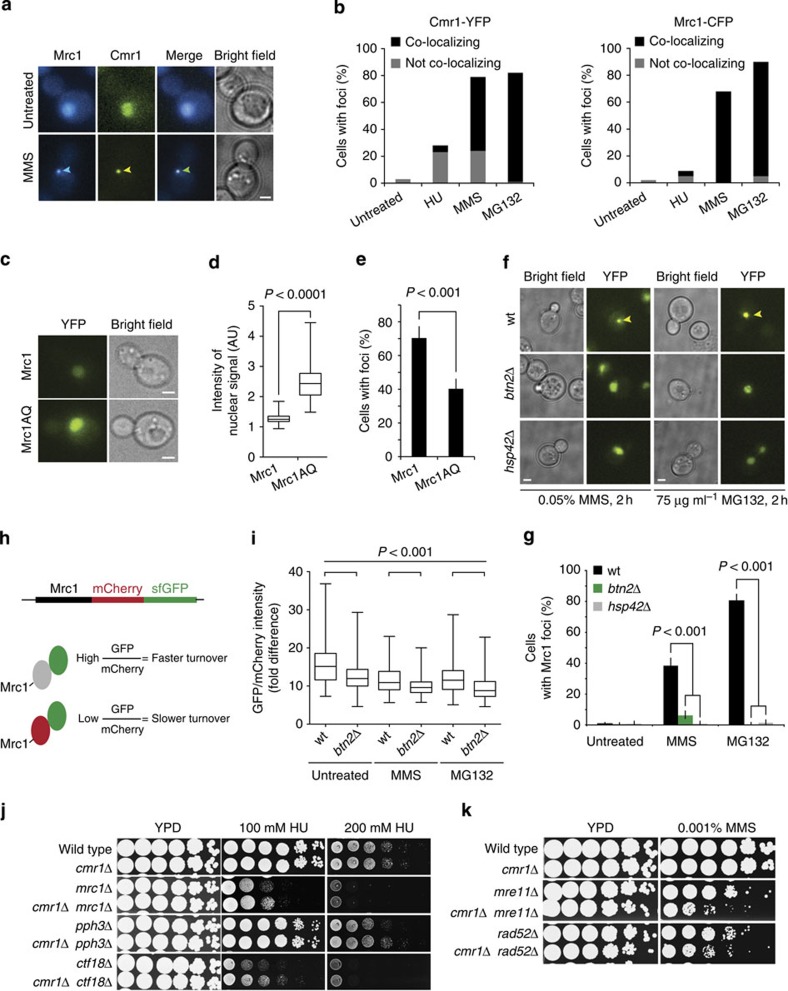
Cmr1 is involved in the response to replication stress. (**a**) Mrc1 and Cmr1 foci co-localize during replication stress. Co-localization between Cmr1-YFP and Mrc1-CFP was assessed in untreated cells (IG160-4A) and after treatment with MMS for 2 h. Representative images are shown. Scale bar, 2 μm. Arrowhead indicates INQ focus. (**b**) Quantification of co-localization of Mrc1 and Cmr1 in response to HU, MMS and MG132 (*n*>200). (**c**) Checkpoint-defective Mrc1AQ protein accumulates in the nucleus. Mrc1-YFP (IG147) and Mrc1AQ-YFP (IG315) were imaged. Scale bars, 2 μm. (**d**) Quantification of Mrc1AQ protein levels. Images from **c** were quantified (*n*>150). The box plot displays fluorescence intensities in arbitrary units (AU), where the line across the box identifies the median sample value, the ends of the box are the 25th and 75th percentiles, and whiskers represent minimum and maximum values. (**e**) Checkpoint-defective Mrc1AQ exhibits reduced recruitment to INQ. Percentage of cells with Mrc1-YFP or Mrc1AQ-YFP foci was quantified after treatment with 0.05% MMS for 2 h. Error bars represent 95% confidence intervals (*n*>150). (**f**) Mrc1 focus formation requires *BTN2* and *HSP42*. Mrc1-YFP localization was assessed in wild-type (IG147), *btn2Δ* (CC1–3B) and *hsp42Δ* (CC2–6B) cells. Representative images of Mrc1 localization are shown. Scale bars, 2 μm. (**g**) Quantification of Mrc1 foci in *hsp42*Δ and *btn2*Δ mutants shown in **f**. Error bars represent 95% confidence intervals. Two replicates, *n*>250. (**h**) Schematic representation of Mrc1 fusion to a fluorescent timer. The ratio between the sfGFP (fast maturing) and mCherry (slow maturing) fluorescence intensities was calculated as a measure of Mrc1 protein turnover. (**i**) Quantification of Mrc1 protein turnover. The fold difference between the GFP and mCherry nuclear fluorescence was measured in wild-type (CC98) and *btn2Δ* (CC102-9C) strains. Box plot were displayed as in **d**. Two replicates, *n*>100. (**j**) *CMR1* deletion suppresses the DNA damage sensitivity of checkpoint mutants. Tenfold serial dilutions were plated on YPD or YPD containing the indicated drug. Strains were ML8–9A (wt), DP1 (*cmr1Δ*), IG156-7D (*mrc1Δ*), IG156-6C (*mrc1Δ cmr1Δ*), IG257-9C (*pph3Δ*), IG257-2C (*pph3Δ cmr1Δ*), IG177-9C (*ctf18Δ*) and IG177-8C (*ctf18Δ cmr1Δ*). (**k**) *cmr1Δ* is synthetic sick with homologous recombination mutants. Tenfold serial dilutions were plated on YPD or YPD containing 0.001% MMS. Strains were ML8-9A (wt), DP1 (*cmr1Δ*), IG164-1D (*mre11Δ*), IG164-2B (*mre11Δ cmr1Δ*), IG162-1D (*rad52Δ*) and IG162-2D (*rad52Δ cmr1Δ*).

Given the interaction of Cmr1 with replication fork components, and based on our observation that the replication checkpoint mediator Mrc1 localizes at INQ (Fig. 3a), we further addressed the functional relationship between Cmr1 and Mrc1. Mrc1-CFP formed foci in response to DNA-damaging agents with a nearly identical profile to that of Cmr1-YFP (Supplementary Fig. 6a), and the two proteins mostly co-localized after MMS or MG132 treatment (Fig. 5a,b). Nevertheless, the ability of Cmr1 or Mrc1 to form foci occurred independently of each other (Supplementary Fig. 6b,c).

Following replication checkpoint activation, Mrc1 is phosphorylated at (S/T)Q sites^2^, which stimulates its degradation via Dia2 and possibly other factors, to promote recovery from replication stress^8^. To establish whether there is a correlation between the degradation of Mrc1 and its relocalization to INQ, we took advantage of the replication-proficient but checkpoint-defective separation-of-function mutant *mrc1AQ*^2^. The *mrc1AQ* mutant showed constitutively higher levels of Mrc1 protein due to a partial defect in Dia2-mediated degradation (Fig. 5c,d)^8^. After treatment with MMS, the Mrc1AQ protein was partially defective in relocalization to INQ (Fig. 5e) and Mrc1 foci were almost completely absent in a *dia2*Δ mutant (Supplementary Fig. 6d). Similarly, abolishing INQ through deletion of *BTN2* or *HSP42* prevented Mrc1 focus formation (Fig. 5f,g) and *btn2*Δ led to a significant decrease in the turnover of Mrc1 with or without replication or proteasomal stress (Fig. 5h,i), as measured using a fluorescence timer construct, consisting of a fast-maturing GFP and a slow-maturing mCherry^26^. Together, these data support a functional relationship between Mrc1 turnover during replication stress and relocalization of the protein to INQ.

### *cmr1*Δ suppresses mutations in *MRC1*, *CTF18* and *PPH3*

In parallel to checkpoint mechanisms, several other pathways contribute to replication stress tolerance including homologous recombination, translesion synthesis, template switching and replication fork stabilization and restart (reviewed in ref. 27), and mutants in these pathways display different degrees of sensitivity to replication stress. The partial redundancy among these pathways may explain the lack of pronounced MMS, HU and ultraviolet irradiation sensitivity of the *cmr1*Δ mutant (see below and ref. 12). Hence, to uncover the epistatic relationship between *CMR1* and known replication stress tolerance pathways, we performed a genome-wide screen for *CMR1* genetic interactions in the presence of replication stress (HU) using the synthetic genetic array (SGA) approach^28^. Differential growth on HU-containing plates between the single and double mutants was assessed using ScreenMill^29^ and revealed negative genetic interactions of *CMR1* with genes in the homologous recombination pathway (*RAD50*, *RAD55* and *MMS4*), and suppression of defects associated with deletion of *MRC1* and *TOF1* of the replication-pausing checkpoint complex (Supplementary Tables 2 and 3). Consistently, *cmr1*Δ additively increased spontaneous chromosome loss in a *rad52*Δ mutant and suppressed the high chromosome loss rates of an *mrc1*Δ mutant (Table 1c). Additional manual testing further showed that deletion of *CMR1* was able to suppress the MMS and HU sensitivity of *pph3*Δ (PP4 phosphatase subunit) and *ctf18*Δ (alternative clamp loader) mutants (Fig. 5j). Importantly, suppression of the MMS sensitivity of the *ctf18Δ* mutant by *cmr1*Δ probably reflected a suppression of the DNA replication checkpoint defect but not the cohesion defects associated with this mutant^30^,^31^,^32^, as the severe defect in sister chromatid cohesion in the *ctf18*Δ mutant was not alleviated by *cmr1*Δ (Supplementary Fig. 7b). Notably, *hsp42*Δ was epistatic with *cmr1*Δ for suppressing the DNA damage sensitivity of *ctf18*Δ, suggesting that relocalization of Cmr1 to INQ is required for Cmr1 function in replication stress tolerance (Supplementary Fig. 7a). Consistent with the increased rate of chromosome loss in the *rad52Δ cmr1*Δ mutant compared with the single mutants, *cmr1*Δ displayed a negative genetic interaction with both *rad52*Δ and *mre11*Δ for survival on MMS (Fig. 5k), indicating a requirement for Cmr1 in the absence of functional homologous recombination. Given that no genetic interactions were observed with genes involved in template switching (*rad5*Δ), MMR (*msh2*Δ, *msh6*Δ and *pms1*Δ), post-replicative repair (*rad18*Δ, *mms2*Δ and *mms22*Δ) or translesion synthesis (*rev3*Δ; Supplementary Fig. 7c–e and Supplementary Table 2), these data suggest that Cmr1 either acts as a negative regulator of a factor required for HU resistance in the absence of Mrc1-Ctf18-Pph3 or promotes a pathway that is toxic in mutants of Mrc1-Ctf18-Pph3.

### Cmr1 promotes DNA-damage checkpoint adaptation

**Figure 6:**
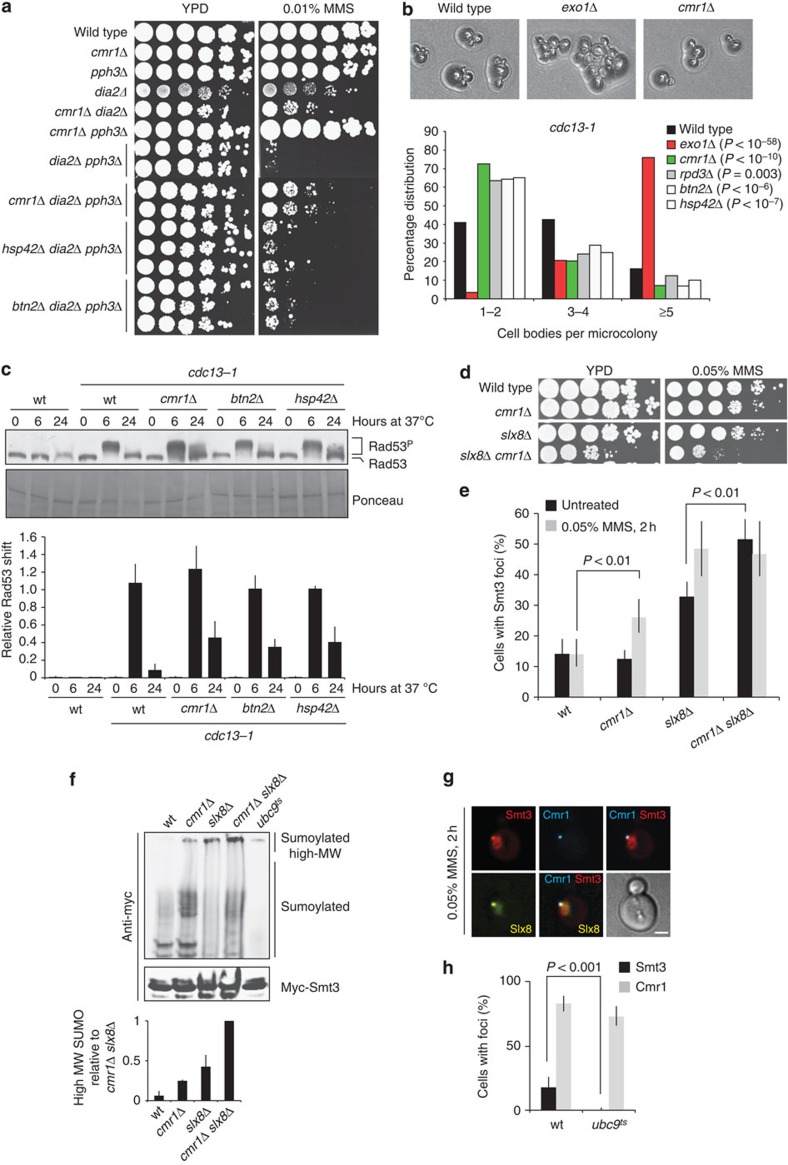
Cmr1 promotes adaptation to the DNA damage checkpoint. (**a**) Cmr1 genetic interaction with checkpoint deactivation pathways is functionally associated with INQ. Wild-type (ML8–9A), *cmr1Δ* (DP1), *dia2Δ* (CC4–19D), *dia2Δ cmr1Δ* (CC4-3B), *pph3Δ* (IG257-9C), *cmr1Δ pph3Δ* (IG257-2C), *dia2Δ pph3Δ* (IG296-2C), *cmr1Δ dia2Δ pph3Δ* (IG296-49B), *hsp42Δ pph3Δ dia2Δ* (IG322-12A) and *btn2Δ pph3Δ dia2Δ* (IG323-19D) strains were plated. (**b**) Mutants of INQ are checkpoint adaptation defective. Strains with a conditional *cdc13-1* mutation and otherwise wild type (ML815-8A), *exo1*Δ (DLY1296), *cmr1*Δ (ML808-12D), *rpd3*Δ (ML807-2D), *btn2*Δ (ML821-4C) or *hsp42*Δ (ML822-4C) were examined. (**c**) Mutants of INQ are defective in Rad53 dephosphorylation during adaptation. *cdc13-1* mutants were grown at 25 °C before being shifted to the restrictive temperature of 37 °C. Samples were harvested at 0, 6 and 24 h after temperature shift. Rad53 phosphorylation was detected by immunoblotting and the relative shift (Rad53^P^/(Rad53+Rad53^P^)) for each of the samples was quantified in the lower panel. Error bars reflect the s.d. of two independent experiments. (**d**) Negative genetic interaction between *cmr1Δ* and *slx8Δ*. Wild-type (ML8–9A), *cmr1Δ* (DP1), *slx8Δ* (NEB290-1B) and *cmr1Δ slx8Δ* (IG256-5D) strains were plated. (**e**) Cmr1 and Slx8 promote turnover of SUMO foci. Wild-type, *cmr1Δ*, *slx8Δ* and *cmr1*Δ *slx8Δ* cells (same strains as in **d**) ectopically expressing yEmRFP-Smt3 (pML133) were imaged. Percentage of cells with Smt3 foci was quantified. Error bars represent 95% confidence intervals (*n*>100). (**f**) Sumoylated and polysumoylated proteins accumulate in the *cmr1Δ* mutant. Cells from the experiment in **d** were transformed with a plasmid expressing 3myc-Smt3 (pRS313-3myc-Smt3), harvested in log phase and immunoprecipitation using anti-myc-coupled dynabeads was performed on whole-cell extracts. The temperature-sensitive *ubc9-1* mutant (IG246-2C) grown at 37 °C was used as a negative control. Bands corresponding to high-molecular weight (high-MW) SUMO conjugates were quantified from two independent experiments using ImageJ. Error bars indicate s.d. (**g**) Co-localization of Slx8 and SUMO at INQ. Cells expressing Cmr1-CFP, Slx8-YFP and yEmRFP-Smt3 (IG302-1D transformed with pML133) were imaged. Representative image of co-localization between Smt3 and Slx8 at INQ (Cmr1) is shown. Scale bar, 2 μm. (**h**) INQ contains sumoylated proteins. Cmr1 and Smt3 localization was monitored in wild type (IG66) and *ubc9*^*ts*^ mutant (IG246-2C) after incubation at 37 °C for 2 h in the presence of 0.05% MMS. Error bars represent 95% confidence intervals (*n*>100).

The negative genetic interaction of *cmr1*Δ with mutations in homologous recombination genes and the suppression of mutations in DNA damage and replication checkpoint genes could be due to Cmr1 promoting replication restart or regulating checkpoint recovery. To directly assess the involvement of Cmr1 in replication fork restart, we released cells from a G1 arrest into S phase in the presence of MMS for 45 min and subsequently monitored completion of DNA synthesis by flow cytometry after MMS removal. As expected, wild-type cells accumulated in S phase in the presence of MMS and slowly recovered from the blockage when the drug was removed (Supplementary Fig. 7f,g). *cmr1Δ* cells were proficient in replication checkpoint activation and restart of DNA synthesis compared with wild type. In contrast, a *dia2*Δ *pph3*Δ mutant was extremely sensitive to MMS and severely defective in replication restart after removal of MMS (Fig. 6a and Supplementary Fig. 7f,g). Notably, *cmr1*Δ partially suppressed the MMS sensitivity of the *dia2*Δ *pph3*Δ mutant without suppressing the replication restart defect, indicating that Cmr1 is not acting directly on replication restart. Moreover, also *btn2*Δ and *hsp42*Δ partially suppressed the MMS sensitivity of the *dia2*Δ *pph3*Δ mutant, suggesting that the suppression conferred by *cmr1*Δ is functionally related to its accumulation at INQ. As Pph3 and Dia2 are regulators of the DNA damage and replication checkpoints, respectively, we sought to test whether the suppression of *dia2*Δ *pph3*Δ MMS sensitivity by *cmr1*Δ is due to a role of Cmr1 in checkpoint adaptation, a mechanism by which cells deactivate the checkpoint after prolonged exposure to DNA damage^33^. To assess the impact of Cmr1 and INQ on the DNA damage checkpoint, we performed a checkpoint adaptation assay using the *cdc13-1* allele, which causes uncapping of telomeres and DNA-damage checkpoint activation at the restrictive temperature^34^. We included in the assay a mutant of the INQ component Rpd3, which has previously been reported to be adaptation defective^35^. After growth at the restrictive temperature for 24 h, the number of cell bodies was counted. This assay indicated that *cmr1*Δ and, to a lesser extent, *rpd3*Δ, *btn2*Δ and *hsp42*Δ are adaptation defective (Fig. 6b). In contrast, an *exo1*Δ mutant, which reduces resection of uncapped telomeres, rescued the *cdc13-1* temperature sensitivity as described previously^36^. Adaptation has previously been linked to Rad53 activity^37^. We therefore examined the electrophoretic mobility shift of phosphorylated Rad53 in the same adaptation assay. *cdc13-1* cells grown at the restrictive temperature for 6 h showed elevated levels of Rad53 phosphorylation, which is completely abolished in the wild type at 24 h, when adaptation has occurred^37^,^38^. Strikingly, Rad53 remained partially phosphorylated at the 24 h time point in the *cmr1*Δ, *btn2*Δ and *hsp42*Δ mutants, which is consistent with the adaptation defect of these mutants (Fig. 6c). Taken together, these data indicate that INQ promotes DNA-damage checkpoint adaptation through attenuation of Rad53 phosphorylation.

### Sumoylated proteins localize at INQ

Sumoylation has previously been implicated in checkpoint regulation^39^ and a strong negative genetic interaction of *cmr1Δ* was observed with mutation of the *SLX5-SLX8* small ubiquitin-like modifier (SUMO)-targeted ubiquitin ligase (STUbL; Fig. 6d and Supplementary Table 2). This raised the possibility that sumoylation could be a signal for proteins to be channelled to the proteasome via INQ and Cmr1. This prompted us to examine the localization of sumoylated proteins in *cmr1Δ* and *slx8Δ* mutant cells. Both single mutants exhibited accumulation of SUMO foci (Fig. 6e), with an additive effect in the *slx8Δ cmr1Δ* double mutant. The increase in SUMO foci in these mutants correlated with the accumulation of high-molecular-weight SUMO-conjugated proteins and this accumulation was more pronounced in the *cmr1Δ slx8*Δ double mutant (Fig. 6f). Rather than being due to increased spontaneous genome instability (Supplementary Fig. 6e), the accumulation of high-molecular-weight SUMO conjugates in the *cmr1Δ* mutant probably reflects a defect in the turnover of the conjugates themselves. Importantly, Slx8 and Cmr1 co-localized with SUMO foci (Fig. 6g), and in the absence of a functional SUMO-conjugating enzyme (*ubc9*^*ts*^) INQ could still form (Cmr1 foci), but SUMO foci were abrogated (Fig. 6h). Altogether, these results suggest that INQ contains sumoylated proteins, and that Cmr1 together with Slx8 facilitate turnover of sumoylated proteins at INQ by the proteasome, molecular chaperones or other mechanisms.

### WDR76 is the orthologue of Cmr1 in higher eukaryotes

**Figure 7:**
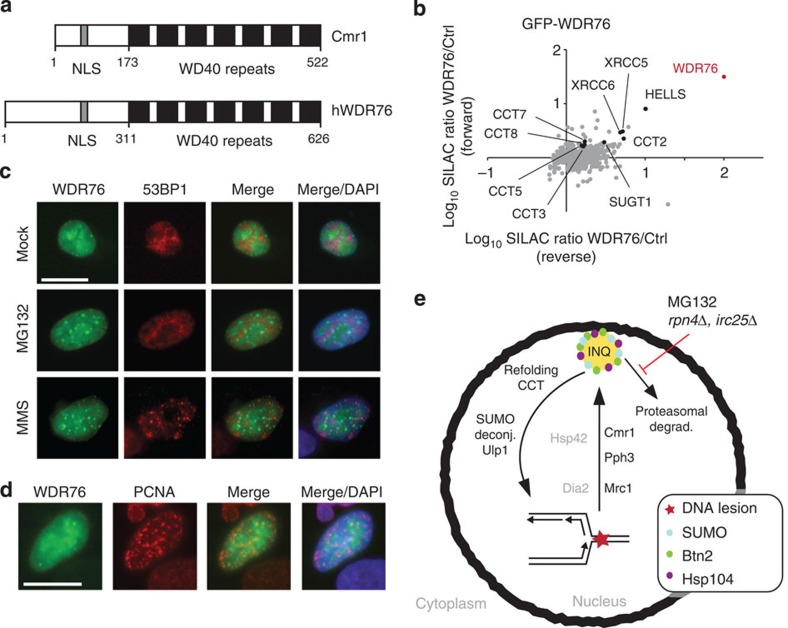
hWDR76 interaction network and subnuclear localization suggest conservation of Cmr1 function. (**a**) Domain organization of Cmr1 and human WDR76. Human WDR76 shares 29% sequence similarity with Cmr1 from *S. cerevisiae*. Filled boxes indicate WD40 repeats. NLS, putative nuclear localization signal. (**b**) Human WDR76 interacts with HELLS, SUGT1, XRCC5, XRCC6 and the CCT–TRiC complex. SILAC-labelled HeLa cells were transfected with GFP-WDR76 or empty vector. GFP-WDR76 and its interacting proteins were enriched using GFP-Trap resin. Proteins were resolved by SDS–PAGE and digested in-gel with trypsin. Peptides were analysed on a quadrupole Orbitrap mass spectrometer. The plot shows log(10) SILAC ratios of proteins associated with GFP-WDR76 compared with background. WDR76 is highlighted in red and several other interactors are also indicated. (**c**) Human WDR76 localizes into nuclear foci. Twenty-four hours after GFP-WDR76 transfection, U2OS cells were treated with 10 μM MG132 or 1.5 mM MMS for 2 h. Immunofluorescence analyses of 53BP1 were performed with anti-53BP1 antibody. DAPI was used to stain nuclei. Scale bar, 20 μm. (**d**) Human WDR76 does not co-localize with PCNA. Stably expressing RFP-PCNA U2OS cells were transfected with GFP-WDR76. 24 h after transfection, cells were fixed and stained with DAPI. Scale bar, 20 μm. (**e**) Model for the role of Cmr1 in promoting replication recovery. See Discussion for details.

WDR76 is the closest orthologue of Cmr1 in higher eukaryotes (Fig. 7a). To evaluate whether the functional characteristics of Cmr1 are conserved in higher eukaryotes, we first identified WDR76 interaction partners by SILAC-based MS analysis. Consistent with data for Cmr1, the top hits of the analysis included subunits of the CCT/TRiC chaperonin (Fig. 7b and Supplementary Table 4). Moreover, two chromatin-related proteins, SUGT1 and HELLS, were identified as WDR76 interactors. In addition, WDR76 was moderately enriched together with SUGT1 and HELLS in nascent chromatin at replication forks in a large-scale proteomic study^40^, suggesting that, similar to Cmr1, WDR76 might have chromatin- and replication-associated functions.

Next we investigated the WDR76 localization using a GFP-WDR76-expressing plasmid. WDR76 associated with chromatin in untreated cells and relocalized into nuclear foci under replication stress conditions (1.5 mM MMS) and after proteasome inhibition (10 μM MG132; Fig. 7c). Consistent with data from yeast, WDR76 did not co-localize with 53BP1 foci, excluding that WDR76 accumulates at the site of DSBs. Moreover, although WDR76 could be detected at some PCNA replication foci, the WDR76 foci did generally not co-localize with replication sites (Fig. 7d), consistent with the observation that Cmr1 is not a constitutive fork component. Taken together, these data suggest a structural and functional conservation of Cmr1/WDR76 in eukaryotes.

## Discussion

Here we characterize a novel stress-induced structure, INQ, which is defined by perinuclear foci of Cmr1 and 27 other yeast proteins, and induced by genotoxic stress and proteasome inhibition. Furthermore, we provide a characterization of the role of Cmr1 in maintenance of genome integrity. Deletion of *CMR1* causes increased chromosome loss and mutation rates, a defect in DNA-damage checkpoint adaptation, and accumulation of sumoylated proteins at INQ. Epistasis analyses for sensitivity to genotoxic stress place *CMR1* upstream of or in parallel to *MRC1*, *CTF18* and *PPH3* in a recombination- and post-replicative repair-independent pathway for genotoxic stress tolerance. Moreover, the negative genetic interaction of *cmr1*Δ with *slx8*Δ is indicative of a role of Cmr1 in recycling or degrading sumoylated proteins either directly or indirectly by promoting DNA repair. The lack of DNA damage sensitivity of the *cmr1*Δ mutant and the wild-type levels of spontaneous Rad52 and Mec1 foci in the untreated condition supports a more direct role of Cmr1 in promoting the desumoylation or degradation of sumoylated proteins. Importantly, many of the phenotypes of *cmr1*Δ are also observed on *btn2*Δ and/or *hsp42*Δ mutation, suggesting that shuttling through INQ constitutes an important aspect of Cmr1 function. However, it remains to be established whether the observed phenotypes of the *btn2*Δ and *hsp42*Δ mutants are directly related to a failure of INQ to form.

The genetic and physical interactions reported for Cmr1 in this study are largely consistent with previous studies, with the addition of the positive genetic interactions that we report here^11^,^13^. With regard to other studies of nuclear foci, we acknowledge that in addition to the 27 Cmr1-co-localizing proteins reported here, other proteins are likely to localize to INQ, given the limited overlap (Apc4, Tub1, Apj1, Hos2 and Dus3) between our genome-wide screen of the GFP strain collection using MG132 and a previous screen of the same collection for nuclear foci induced by MMS, which identified 28 proteins^13^. Some of the factors that form nuclear foci after MMS treatment are DNA repair proteins, which we show do not co-localize with INQ. Moreover, MMS-induced Hsp104, Mkt1, Ylr126C and Gln1 foci have been annotated as cytosolic in the previous study^13^, although we find that a subset of these foci are in fact nuclear and co-localize with Cmr1.

Importantly, although Cmr1 and VHL foci co-localize in the nucleus, they exhibit different genetic requirements. Although Cmr1 foci require both *BTN2* and *HSP42*, the relocalization of misfolded VHL to INQ only depends on *BTN2* (Supplementary Fig. 4b). Interestingly, VHL foci can also be induced by MMS, although less efficiently, and these foci are exclusively nuclear and require both *BTN2* and *HSP42*. These observations indicate that the stress caused by MMS is primarily nuclear and point to functional differences between Btn2 and Hsp42, depending on the type of stress. In particular, being a component of INQ, Btn2 is likely to be structurally involved in the formation of this nuclear compartment, whereas Hsp42 might regulate the relocalization of INQ substrates indirectly, particularly in response to replication stress. Altogether, the differences in genetic requirements and the variety of functions among its components define INQ as a multifunctional compartment gathering different kinds of substrates, only a proportion of which are misfolded proteins. The possible existence of different types of Cmr1 foci is also indicated by a subset of Cmr1 foci lacking Mrc1 (Fig. 5b). To determine the full spectrum of biological processes involving INQ, we believe that it will be important to examine protein relocalization to INQ in other stress conditions such as ultraviolet irradiation, heat shock and nutrient starvation.

As reported previously^11^,^12^, we find that Cmr1 is a constitutive component of chromatin and interacts with DNA replication factors such as the MCM helicase and subunits of the replication factor C clamp loader (Fig. 4 and Supplementary Fig. 5). We believe these interactions to be transient, induced by replication stress and possibly mediated by posttranslational modifications. Moreover, given that Cmr1 has been shown to bind to DNA *in vitro*^12^ and co-purifies with histones^11^, it is conceivable that the association with chromatin could be achieved by direct physical interaction with DNA or nucleosomes, probably through the WD40 domain. Consistently, chromatin immunoprecipitation of Cmr1 and the replicative polymerase (Pol2) showed that Cmr1 binds chromatin independently of DNA replication (Supplementary Fig. 8a–c). In line with a recent view on the regulation of the removal and turnover of sumoylated protein complexes by proteasome-dependent degradation pathways^41^, our data raise the possibility of Cmr1 being readily available on chromatin, to promote turnover of phosphorylated, ubiquitylated or sumoylated targets from stalled replication forks, thereby facilitating an efficient response to replication stress (Fig. 7e). Based on our model, this process involves INQ, in line with our establishment of a functional relationship between the ability of Cmr1 to accumulate at INQ and its role in genome maintenance. This relationship does not seem to be restricted to Cmr1 as documented by the similar accumulation of Mrc1 at INQ. In the case of Mrc1, relocalization to INQ requires Dia2, indicating that Mrc1 ubiquitylation is required. Consistently, we observed a correlation between Mrc1 protein levels and its relocalization to INQ in *mrc1AQ* and *btn2Δ* mutants (Fig. 5c–g), respectively, which is consistent with the reduced Mrc1 protein turnover observed when INQ is abolished by deletion of *BTN2* (Fig. 5h,i). As exemplified by Mrc1, we hypothesize that each INQ-targeted substrate will require specific mediators for their relocalization. Moreover, the accumulation of proteins at INQ on inhibition or mutation of the proteasome and/or the Slx5-Slx8 STUbL indicates that some INQ-targeted proteins are substrates of SUMO-dependent degradation.

Previous studies have described several classes of stress-induced cytoplasmic foci for protein aggregation including the JUNQ compartment and the IPOD compartment (for review see refs 42, 43). JUNQ is formed by misfolded proteins, which are normally degraded in a manner dependent on chaperones and the ubiquitin proteasome system^20^,^21^,^22^. In this study, we present evidence that a JUNQ-like structure (INQ) can form in the nucleus in response to DNA replication stress and proteasome inhibition. This conclusion is based on several lines of evidence. First, INQ localizes inside the nucleus, using as reference two independent markers of the nuclear periphery, Nup49 and Hmg1, where Hmg1 localizes continuously throughout the nuclear envelope (Supplementary Fig. 2a). Second, >95% of nuclear foci of misfolded VHL co-localized with Cmr1, while none of the juxtanuclear or cytoplasmic VHL foci contain Cmr1 (Fig. 3d,e). Third, MMS induced exclusively nuclear VHL foci, suggesting that in response to DNA replication stress VHL is recruited to INQ. Finally, the majority of the INQ-localized proteins are exclusively nuclear (67%, 18/27) in the absence of stress. We propose that the remaining proteins that do not generally localize to the nucleus in the absence of stress might be translocated to the nucleus specifically during stress, or that a minor nuclear pool of these proteins could become detectable on relocalization to INQ due to the increased local concentration.

Similar to Cmr1, its closest human orthologue WDR76 relocalizes from a diffuse nuclear distribution to distinct subnuclear foci in response to MMS and MG132. Moreover, WDR76 interacts with chromatin components and the CCT chaperonin. Although we have not further investigated the nature of WDR76 foci, the composition and properties of these structures raise the possibility of a functional similarity between INQ in yeast and promyelocytic leukemia (PML) nuclear bodies in higher eukaryotes. In particular, promyelocytic leukemia nuclear bodies are induced in number and size by genotoxic stress and proteasome inhibition^44^,^45^, they contain both poly-sumoylated species and the RNF4 STUbL^46^, and they appear to play a role in chromatin-associated processes^47^. Whether WDR76 directly participates in the replication stress response remains to be addressed.

Finally, the diverse set of proteins that localize to INQ suggests that other biological processes may be regulated through this structure. For example, several components of the anaphase-promoting complex (Cdc20, Cdc27 and Apc4), which targets substrates for proteasomal degradation during the metaphase-to-anaphase transition (reviewed in ref. 48), localize to INQ, suggesting that cell cycle progression during mitosis may also require shuttling of key factors through INQ. Some INQ localizing factors such as Gln1 and Dus3 have no reported link to maintenance of genome integrity. However, Gln1 function is linked to nutrient starvation and both proteins were reported to provide resistance to osmotic stress, suggesting that different stress responses could be coordinated at INQ or use similar signalling mechanisms^49^,^50^,^51^,^52^. Future studies will be aimed at dissecting the mechanisms that promote relocalization and, possibly, turnover of individual proteins through INQ.

## Methods

### Yeast strains and cell culture

Standard media were used throughout this study^53^. Standard genetic techniques were used to manipulate yeast strains^53^. Unless otherwise stated, yeast strains used in this study are *RAD5* derivatives of W303 (Supplementary Table 5). Strains from the GFP-fusion library (Invitrogen), gene disruption collection (Invitrogen) and VN-fusion collection (Bioneer) are derivatives of S288C. Exceptions and mixed backgrounds are indicated.

### Yeast constructs and plasmids

Construction of fluorescently tagged proteins was performed using adaptamer-mediated PCR^54^. To obtain the *CMR1-VC::KanMX6* construct, a PCR product containing *VC-T*_*ADH1*_*-KanMX6* was amplified from pFA6a-VC155 (Bioneer), using Cmr1-VC155-fw and Cmr1-VC155-rv primers adapted with overhangs, to target integration of the construct at the C-terminal end of *CMR1*. The PCR product was transformed into ML659-4B, expressing *NLS-yEmRFP::HIS3* to produce IG241. To generate *CMR1-YFP::NatMX*, the *NatMX* cassette from p4339 was amplified using Cmr1-YFP::NatMX-fw and Cmr1-YFP::NatMX-rv primers, and the fragment targeted downstream of *CMR1-YFP* terminator in IG66 to give IG188.

To generate plasmids pIG13, pIG14 and pIG15, PCR fragments containing C-terminal YFP fusions of full-length *CMR1-YFP*, *CMR1-NTD*_*(1–17*3)_*-YFP* or *CMR1-NLS-WD40*_*(174–522)*_*-YFP*, respectively, including the endogenous promoter and terminator flanked by HindIII and XhoI sites, were cloned into HindIII/XhoI-linearized pRS426. *CMR1-YFP* fragment was amplified from genomic DNA extracted from IG66, using Cmr1up-F_HindIII and Cmr1down-R_XhoI primers. *CMR1-NTD*_*(1–17*3)_ was created by fusion PCR between two fragments amplified from pIG13. The first fragment, including the promoter region and the NTD of *CMR1*, was amplified with Cmr1-up-F_HindIII and Cmr1N-term-rv primers, while the second fragment containing YFP and the terminator region was amplified with Cmr1N-term-fw (harbouring the complementary sequence for annealing with the first fragment) and Cmr1down-R_XhoI. Fusion PCR using the two fragments as template was performed with Cmr1upF_HindIII and Cmr1down-R_XhoI primers. *CMR1-NLS-WD40*_*(174–522*)_ was created with a similar approach. The first fragment, including the promoter region of *CMR1* until the START codon, was amplified with Cmr1up-F_HindIII and Cmr1WD40-rv (harbouring the complementary sequence for annealing with the second fragment), while the second fragment containing the WD40 domain fused to YFP and the terminator region was amplified with Cmr1WD40-fw and Cmr1down-R_XhoI. Cmr1WD40-fw contained an ATG and a sequence encoding SV40-NLS (PKKKRKVEDP).

All the subunits (Cct1 to Cct8) are essential for survival in eukaryotes and live-cell visualization of the CCT complex had not been successful so far. We took advantage of the crystal structure of the yeast CCT^55^,^56^, to introduce a YFP tag into a loop of Cct6 (between position P373 and K374), which is predicted to lie on the outer surface of the complex^57^. pGP564 (clone YGPM23c07) from the yeast genomic tiling array collection (Thermo Scientific) was linearized by digestion with BlpI, which cuts in *CCT6*, and gap repaired by co-transformation with a fusion PCR fragment containing a partial sequence of *CCT6* spanning over the BlpI restriction site, with YFP inserted between P373 and K374 into strain ML8–9A. This PCR fragment was obtained by fusion of three overlapping PCR products generated using primers CCT6_1-fw, CCT6_2-rv, CCT6_3-fw, CCT6_4-rv, CCT6_5-fw and CCT6_6-rv, and pGP564 and pWJ1165 as templates. The resulting CCT6-YFP-containing plasmid was named pIG20.

The high-copy plasmid pML84, used for ectopic expression of an NLS-RFP fusion as a nuclear marker, was constructed by first amplifying *yEmRFP* from pNEB30 using KpnI and EcoRI-adapted primers NLSyEmRFP-F and NLSyEmRFP-R, respectively, that adds the SV40-NLS (PKKKRKVEDP) to the N-terminal end of yEmRFP. The KpnI/EcoRI-digested PCR product was cloned into KpnI/EcoRI-linearized pGAD-C2 behind the *ADH1* promoter to generate pML84.

The Cmr1–7Myc::KanMX6 strain was constructed by PCR-based tagging, using primers Cmr1–13mycFW, Cmr1–13mycRV and pFA6a as template.

To construct a single-copy plasmid pML133 for expression of yEmRFP-Smt3, *yEmRFP* was first integrated into the native *SMT3* locus of a diploid strain prepared by mating IG66 and W4700-10C. The *yEmRFP* was integrated immediately before the start codon of *SMT3* along with the sequence 5′- GGAGGTCCAGGTGGA -3′ encoding a GGPGG linker using PCR-based allele replacement^58^. In brief, the homology arms for targeted integration were generated from template genomic DNA using primers Smt3-up-F, yEmRFP-Smt3-up-R, yEmRFP-Smt3start-F and SMT3-R. Next, the two homology arms were fused by PCR to DNA sequences containing *yEmRFP* joined to either the 5′- or 3′-end of *K.l. URA3* that were PCR amplified by primers Cherry.Fw and 3′-int or 5′-int and yEmRFP-R from vectors pNEB30 and pNEB31, respectively. The two PCR fusion products were co-transformed into yeast and transformants selected on SC-Ura. After pop-out of the *K.l. URA3* marker by selection on 5-FOA, the genomic *yEmRFP-SMT3* locus was sequenced and the diploid strain was sporulated to obtain a haploid strain ML702-R expressing yEmRFP-Smt3. Next, the yEmRFP-Smt3 fusion was gap repaired onto BlpI-linearized pGP564 (clone YGPM25o09) from the yeast genomic tiling array collection (Thermo Scientific), to generate plasmid pML122. Finally, a ClaI restriction fragment of pML122 containing the *yEmRFP-SMT3* fusion was subcloned into the ClaI site of pRS416 to produce pML133.

For construction of a strain with triple-tagged Cmr1–3 × YFP, we first assembled a fusion of either the 5′- or 3′-end of *K.l. URA3* with a triple array of YFP into pWJ1164 and pWJ1165, to produce plasmids pML97 and pML98, respectively. First, YFP was PCR amplified from pWJ1164 using adapted primers 6ala-F-ClaI and XFP-R-EcoRI, digested with ClaI and EcoRI, and cloned into ClaI/EcoRI-digested pWJ1164. Second, a EcoRI/XmaI-adapted YFP fragment generated by PCR using primers XFP-F-EcoRI and XFP-R-XmaI, and pWJ1164 as a template, was cloned into the EcoRI/XmaI site of this vector to produce pML97. Similarly, YFP was PCR amplified from pWJ1165 using adapted primers XFP-F-SacI and XFP-R-StuI-SphI-SacI, digested with SacI and cloned into SacI-linearized pWJ1165. Second, a StuI/SphI-adapted YFP fragment generated by PCR using primers XFP-F-StuI and XFPstop-R-SphI, and pWJ1165 as a template, was cloned into the StuI/SphI site of this vector to produce pML98. The triple YFP (3 × YFP) was integrated immediately before the stop codon of *CMR1* along with a sequence encoding a 6-alanine linker using PCR-based allele replacement^58^. In brief, the homology arms for targeted integration were generated from template genomic DNA using primers Cmr1-F, Cmr1–6ala-up, Cmr1–3xdown and Cmr1-down-R. Next, the two homology arms were fused by PCR to DNA sequences containing 3 × YFP joined to either the 5′- or 3′-end of *K.l. URA3* that were PCR amplified by primers 6ala-F and 3′-int or 5′-int, and term-R from vectors pML97 and pML98, respectively. The two PCR fusion products were co-transformed into yeast and transformants selected on SC-Ura. After pop-out of the *K.l. URA3* marker by selection on 5-FOA, the presence of three copies of YFP was confirmed by PCR.

To generate the Mrc1 timer construct, the mCherry-sfGFP::hphNT1 cassette of pMaM60 (ref. 26) was amplified using Mrc1-S3-F and Mrc1-S2-R primers adapted with overhangs, to target integration of the construct at the C-terminal end of *MRC1*. The PCR product was transformed into ML8–9A to create CC98.

Plasmids are described in Supplementary Table 6. Oligonucleotide sequences are listed in Supplementary Table 7.

### SILAC and MS analysis

For identification of proteins interacting with Rad52, Rfa1 and Cmr1, *S. cerevisiae* cells from lysine auxotroph strains expressing Rad52-YFP, Rfa1-YFP and Cmr1-YFP fusions, respectively, were grown in synthetic complete medium containing lysine0 (^12^C_6_ and ^14^N_2_) or lysine8 (^13^C_6_ and ^15^N_2_; Sigma, 608041) for more than ten generations. Cultures were treated with 200 μg ml^−1^ zeocin (Invitrogen) for 2 h (Rad52-YFP) or 0.03% MMS (Sigma) for 2 h (Rfa1-YFP and Cmr1-YFP), and 50 optical densities per culture were harvested. Proteins were extracted in lysis buffer without EDTA (10 mM Tris-HCl pH 7.5, 150 mM NaCl, 0.5% NP40, 1 mM phenylmethyl sulphonyl fluoride, 1 × complete protease inhibitor (Roche)) and whole-cell extracts were incubated with 25 μl of equilibrated GFP-Trap_A beads (Chromotek) for 2 h at 4 °C. Beads were washed in 50 volumes of ice-cold dilution buffer (10 mM Tris-HCl pH 7.5, 150 mM NaCl, 1 mM phenylmethyl sulphonyl fluoride, 1 × complete protease inhibitor (Roche)), before beads incubated with heavy-labelled and light-labelled proteins were mixed and washed in 10 volumes dilution buffer. For releasing proteins from the beads, samples containing immunoprecipitated proteins were incubated with one volume of 4 × LDS Sample Buffer (NuPAGE), 40 mM dithiothreitol for 10 min at 70 °C and then at room temperature for 35 min. Chloroacetamide was added to a final concentration of 110 mM and samples incubated at room temperature for 45 min. Proteins were resolved by SDS–PAGE and each loaded gel lane was sliced into three or four slices containing an estimated equal amount of proteins. Gel pieces were in-gel digested with trypsin protease (13 ng μl^−1^ trypsin in 20 mM NH_4_HCO_3_). In-gel trypsin digestion was carried out overnight at 37 °C, peptides were extracted by incubating the gel pieces with increasing concentration of acetonitrile and collecting the resulting fractions. The organic solvent was removed by vacuum centrifugation and peptides were reconstituted in acidified water (containing 0.5% acetic acid). Peptides were purified with reversed-phase C18 packed StageTips^59^. StageTips were activated with methanol and equilibrated with 0.5% acetic acid before loading the peptides, and washed twice with 5% acetonitrile, 0.5% acetic acid. Immediately before MS analysis, peptides were eluted from StageTips with 40 μl 60% acetonitrile, 0.5% acetic acid, sample volume was reduced to ~4 μl by vacuum centrifugation and the samples were acidified with 2 μl of 0.5% acetic acid, 1% trifluoroacetic acid solution. The samples were analysed by liquid chromatography–MS/MS using the EASY-nLC HPLC (Thermo Scientific) and quadrupole Orbitrap mass spectrometers (LTQ-Orbitrap Velos or Q-Exactive, Thermo Scientific). Samples were loaded on 15-cm long reversed-phase columns (diameter 75 μm, packed with 3 μm size C18-AQ material (ReproSil-Pur, Dr Maisch). Peptides were separated by using a linear gradient of acetonitrile (from 5% to 40%) and 0.5% acetic acid. Mass spectrometers were operated in a positive ion mode, data-dependent manner, automatically switching between MS and MS^2^ acquisition. The survey full scan was set up to scan *m*/*z*=300–1,700 for the Velos mass spectrometer and *m*/*z*=300–1,750 for the Q Exactive mass spectrometer, with resolutions of 30,000 and 70,000 for the Velos and Q Exactive, respectively. The top 10 or 12 most intense ions were sequentially isolated and fragmented by higher-energy C-trap dissociation. An ion selection threshold of 5,000 was used. Peptides with unassigned charge states, as well as with charge state <+2 were excluded from fragmentation. A dynamic exclusion window of 30 s was used to limit re-sequencing. Fragment spectra were acquired in the Orbitrap mass analyser. MS raw data files were analysed with the MaxQuant software package (developer’s version 1.2.2.9)^60^,^61^. Full-scan peaks and fragment-scan peaks were searched against the Saccharomyces Genome Database release 63 containing 6,717 putative protein sequences (http://downloads.yeastgenome.org/). Standard settings were used for the software, except that ‘Minimum Ratio Count’ was set to 1. Cysteine carbamidomethylation was searched as a fixed modification, whereas protein N-terminal acetylation and methionine oxidation were searched as variable modifications. Database search was performed with a mass tolerance of 6 p.p.m. for precursor ions and 20 p.p.m. for fragment ions. False discovery rate was estimated using a target-decoy approach, allowing a maximum of 1% false identifications from the reversed-sequence database.

For determining WDR76 interactome, HeLa cells were cultured in DMEM medium supplemented with 10% fetal bovine serum, L-glutamine, penicillin and streptomycin. For SILAC labelling, cells were cultured in media containing either 84 μg ml^−1^ L-arginine and 146 μg ml^−1^ L-lysine or 84 μg ml^−1^ L-arginine-U-^13^C_6_-^15^N_4_ and 146 μg ml^−1^ L-lysine-U-^13^C_6_-^15^N_2_ (Cambridge Isotope Laboratories) for ~14 days^62^. Cells were transfected with pcDNA-DEST53-GFP-WDR76 or empty vector and lysed in modified RIPA buffer (50 mM Tris-HCl pH 7.5, 150 mM NaCl, 1% NP-40, 0.1% Na-deoxycholate, 1 mM EDTA and protease inhibitors 5 mM β-glycerophosphate, 5 mM NaF, 1 mM Na-orthovanadate, complete protease inhibitor cocktail (Roche)) after 48 h. Lysates were cleared by centrifugation at 17,000*g* for 15 min at 4 °C, and GFP-WDR76 and its interacting proteins were enriched using GFP-Trap resin (ChromoTek) for 2 h. Proteins were resolved by SDS–PAGE and digested in-gel with trypsin. Peptide fractions were analysed on a quadrupole Orbitrap mass spectrometer (Q-Exactive, Thermo Scientific) equipped with a nanoflow HPLC system (Thermo Scientific)^63^. Raw data files were analysed using MaxQuant software (version 1.2.2.9)^60^,^61^.

### Synthetic genetic array

SGA technology^64^ was used to transfer the *CMR1-VC::KanMX6* and the *CMR1-YFP::NatMX NLS-yEmRFP::URA3* constructs from the query strains (IG241 and IG189-10B, respectively) to each of the VN fusion library (Bioneer) and gene deletion collection (Invitrogen) strains, respectively. For analysis of *CMR1* genetic interactions, SGA analysis was performed in quadruplicates (1,536 format). *MAT***a** meiotic progeny derived from the *cmr1::NatMX* query strain (IG105) or the control strain (SG936) were tested for viability on YPD plates containing 200 mM HU. Plates were scanned (ScanMaker 9800XL, Microtek) and growth differences between the tester and control plates were quantified using ScreenMill^29^.

### Yeast live-cell imaging and immunofluorescence

For live-cell imaging, cells were grown at 25 °C in synthetic complete or the appropriate dropout medium supplemented with adenine (100 μg ml^−1^), unless otherwise stated. For detection of untagged Cmr1 by immunofluorescence, fixed cells were incubated with anti-Cmr1 primary antibody (kind gift of Sung-Ho Bae)^12^, followed by staining with Alexa Fluor 594-conjugated anti-rabbit secondary antibody at 1:1,000 dilution (Invitrogen, catalogue number A11037). DNA was stained by adding 10 μg ml^−1^ DAPI (4',6-diamidino-2-phenylindole). Fluorophores were visualized on a DeltaVision Elite microscope (Applied Precision, Inc.). For statistics, at least 100 morphologically intact cells were examined. Fluorescence intensities were measured with Volocity software (PerkinElmer) and presented as box plots using Prism software (GraphPad Software, Inc.).

For high-throughput fluorescence microscopy, cells were grown in 96-well plates in synthetic complete medium supplemented with adenine (100 μg ml^−1^) and diluted 20-fold before imaging in 384-well CellCarrier plates (PerkinElmer). Drug treatment was performed manually or by automated dispensing. Imaging was performed on an Opera QEHS high-content screening microscope (PerkinElmer). For the GFP co-localization screen, stacked images of five fields per well were acquired. One second exposure time for each channel was used. For the Cmr1-YFP screens, ten fields per well were acquired. One second exposure time was used. Data analysis was performed using Columbus software (PerkinElmer).

### Microscopy of human cells

Cells were pre-extracted with 0.5% Triton in CSK buffer (10 mM PIPES pH 7, 100 mM NaCl, 300 mM sucrose, 3 mM MgCl_2_) for 5 min at 4 °C before fixation or fixed directly with 4% formaldehyde. The cells were blocked with 5% BSA in PBS-T, incubated with primary antibody, washed three times with PBS-T, incubated with secondary antibody and washed again. DNA was counterstained with DAPI and slides were mounted using Vectashield anti-fade (Vector)^65^. Primary antibodies against 53BP1 (NB100–904, Novus) at 1:200 dilution and secondary Alexa Fluor-coupled antibodies (A11037, Invitrogen) at 1:1,000 dilution were commercial. U2OS cells stably expressing RFP-PCNA were described^66^. Images were collected using a DeltaVision system.

### DNA damage sensitivity

For analysis of drug sensitivity on solid medium, tenfold serial dilutions were prepared from a saturated overnight culture. Zeocin (Invitrogen), MMS (Sigma), HU (Sigma), 4-nitroquinoline-1-oxide (Sigma) and camptothecin (Sigma) were added to the final concentrations stated in the figure legends. After spotting, the plates were incubated at 30 °C for 2–4 days.

### Protein analysis

For detection of Cmr1 protein levels, whole-cell extracts were obtained by TCA extraction. Frozen cell pellets were resuspended in 20% TCA and beaten with glass beads. Beads were washed twice in 5% TCA and the washes combined with the lysed cell mix to yield a final concentration of 10% TCA. The cell extract was centrifuged, the pellet resuspended in Laemmli buffer and neutralized with 1 M Tris-base solution. Samples were boiled, centrifuged and the supernatant loaded on polyacrylamide gels. Rabbit anti-Cmr1 (1:250, clone 2.1, Cambridge Research Biochemicals) followed by horseradish peroxidase-conjugated swine anti-rabbit antibody (1:5,000, P0399, Dako) was used for detecting Cmr1-TAP. Tubulin was detected using rat anti-tubulin (1:5,000, ab6160, abcam) followed by horseradish peroxidase-conjugated rabbit anti-rat (1:5,000, P0450, Dako). For detection of sumoylated proteins, cells expressing 3myc-Smt3 were harvested in log phase and proteins were extracted by bead beating in extraction buffer (10 mM Tris-HCl pH 7.5, 0.5% NP-40, 150 mM NaCl). *N*-ethylmaleimide (50 mM) was added during the extraction and immunoprecipitation, to stabilize SUMO-conjugates. For immunoprecipitation, monoclonal anti-Myc antibody (9E10, Santa Cruz Biotechnology, catalogue number sc-40) was incubated with whole-cell extracts before coupling with equilibrated Dynabeads. Rad53 phosphorylation was detected using an anti-Rad53 antibody (Abcam, catalogue number ab104232) at 1:2,000 dilution. Uncropped immunoblots are shown in Supplementary Fig. 9.

### Chromosome loss and mutation rates

The rate of spontaneous mutation at *CAN1* resulting in canavanine resistance (Can^R^) was determined by plating overnight stationary cultures onto synthetic complete medium lacking arginine and supplemented with L-canavanine (50 μg ml^−1^). To calculate the rate of spontaneous frameshift mutations at the *lys2::InsA*_*14*_ locus, cells were plated on synthetic complete medium lacking lysine^67^. Five to twenty-one single cultures were analysed for each strain and the median frequency was used to determine the mutation rate^68^. To determine the replication stress-induced mutation frequencies, overnight cultures were diluted and exposed to MMS before plating.

Rates of loss of the mating-type (*MAT*) locus on chromosome *III* were determined as a measure of chromosome loss by the bimaters assay. Overnight cultures of wild-type or homozygous mutant diploid strains were appropriately diluted, plated onto complete medium and grown for 1–2 days. Cells were subsequently tested for the ability to mate with a *MAT***a** tester strain (R113) and mating events deriving from the loss of the *MAT***a** loci were counted. Three to seventeen trials were analysed and the s.d. was used to compare the different mutants.

### Checkpoint adaptation assay

The *cdc13-1* mutation was introduced into relevant mutant strains by genetic crossing. To measure checkpoint adaptation^34^, cells were grown shaking in YPD to mid-log phase, shifted to 32 °C for 2 h, sonicated, diluted appropriately and spread onto preheated YPD plates at 32 °C. After incubation of the plates at 32 °C for 24 h, images were acquired of 200–300 microcolonies and the number of cell bodies in each colony counted.

### Statistical methods

For microscopy experiments, the significance of the differences observed between different cell populations was determined by one-tailed Fisher’s exact test. *P*-values with *P*<0.05 were considered significant. The 95% confidence interval for the median of a population was used to compare mutation rates.

## Author contributions

I.G., C. Colding, S.S. and M.L. conducted the majority of the yeast experiments. P.H., P.B. and C. Choudhary performed MS-based protein–protein interaction analysis. K.N. and A.G. conducted microscopy of human cells. J.O. and E.H. performed the yeast screen for mutants that affect mutation rates. D.P.M. constructed strains. All authors contributed to designing experiments and writing the manuscript.

## Additional information

**How to cite this article:** Gallina, I. *et al*. Cmr1/WDR76 defines a nuclear genotoxic stress body linking genome integrity and protein quality control. *Nat. Commun.* 6:6533 doi: 10.1038/ncomms7533 (2015).

## Supplementary Material

Supplementary Information Supplementary Figures 1-9, Supplementary Tables 1-7, Supplementary Methods and Supplementary References

Supplementary Data 1 List of protein-protein interactions from Cmr1 SILAC experiment. Identified proteins with more than 1.5-fold difference between both the H/L and L/H normalized ratios for the two independent Cmr1-YFP pull down experiments are reported in the accompanying Excel sheet. Cmr1 is highlighted in yellow. Hits are ranked by the normalized H/L ratio.

## Figures and Tables

**Table 1:** Effect of Cmr1 on mutation rates and chromosome loss.

**a. Mutation rates**			
**Genotype**	**Strain**	Mutation rate (fold change*)	
		**Can**^**R**^ **(× 10**^**−7**^**)**	**Lys**^**+**^ **(× 10**^**−7**^**)**
Wild type	IG106-4D	3.8	4.5
*cmr1Δ*	IG106-1C	4.7 (1.2)	16 (4)†
*mlh1Δ*	IG106-5A	63 (17)†	77420 (17204)†
*cmr1Δ mlh1Δ*	IG106-1D	91 (24)†	23790 (5278)†‡
*msh2Δ*	IG137- 66D	56 (15)†	30950 (6878)†
*cmr1Δ msh2Δ*	IG137-28C	338 (89)†‡	43530 (9673)†
*mrc1Δ*	IG172-7C	16 (4)†	6.2 (1.4)
*cmr1Δ mrc1Δ*	IG172-4B	21 (6)†	6.9 (1.5)

**b. Replication stress-induced mutation frequencies**			
**Genotype**	**Strain**	Mutation frequency (fold change*)	
		**Can**^**R**^ **(× 10**^**−7**^**)**	**Lys**^**+**^ **(× 10**^**−7**^**)**
Wild type	IG106-4D	53	40
*cmr1Δ*	IG106-1C	34 (0.6)	99 (2.5)†

**c. Rates of chromosome loss (BiM assay)**^70^			
**Genotype**	**Strains**	**Chromosome loss (× 10**^−2^) (fold change*)	**s.d. (× 10**^−2^**)**
Wild type	ML8-9A × W4700-10C	3.3	0.5
*cmr1Δ*	IG79-6D × DP1	6.6 (2)†	1.1
*rad52Δ*	SMG259-3C × SMG259-11B	7.5 (2.3)†	1.6
*cmr1Δ rad52Δ*	IG162-2D × IG184-11C	14.5 (4.5)†‡	3.3
Wild type	ML8-9A × W4700-10C	0.4	0.09
*cmr1Δ*	IG79-6D × DP1	0.8 (2.1)	0.7
*mrc1Δ*	IG156-7D × IG179-3B	3.1 (7.8)†	0.8
*cmr1Δ mrc1Δ*	IG156-9B × IG156-9C	1.9 (4.8)†	0.5

BiM, bimaters assay; MMS, methyl methanesulfonate.

(**a**) Spontaneous mutation rates. Forward (Can^R^) and frameshift (*LYS2*_14A_) mutation rates were determined for IG106-4D (wt), IG106-1C (*cmr1Δ*), IG106-5A (*mlh1Δ*), IG106-1D (*cmr1Δ mlh1Δ*), IG137-66D (*msh2Δ*), IG137-28C (*msh2Δ cmr1Δ*), IG172-7C (*mrc1Δ*) and IG172-4B (*mrc1Δ cmr1Δ*). (**b**) Induced mutation frequencies. Fold change compared with wild type is indicated in parentheses. Replication stress-induced mutation frequencies were determined after growth in 0.03% MMS for 30 min. (**c**) Rates of chromosome loss (BiM assay). Diploid strains homozygous for the indicated gene deletions were grown on rich medium and subsequently tested for the ability to mate with a *MAT***a** tester strain (R113). Frequency of mating, derived from loss of the endogenous *MAT***a** locus, is used as a measure of chromosomal instability. The two sets of BiM assays were performed with different batches of media; s.d. of the rate is reported.

^*^Relative to wild type.

^†^Significant (*P*<0.05) compared with wild type.

^‡^Significant (*P*<0.05) difference between single and double mutant.
