# Supplementary material for: Cmr1/WDR76 defines a nuclear genotoxic stress body linking genome integrity and protein quality control
Source: Nat Commun. 2015 Mar 30;6:6533. doi: 10.1038/ncomms7533 (PMC4389229; doi:10.1038/ncomms7533)
Supplement: Supplementary Information — Supplementary Figures 1-9, Supplementary Tables 1-7, Supplementary Methods and Supplementary References [file ncomms7533-s1.pdf]

SUPPLEMENTARY INFORMATION

Supplementary figures

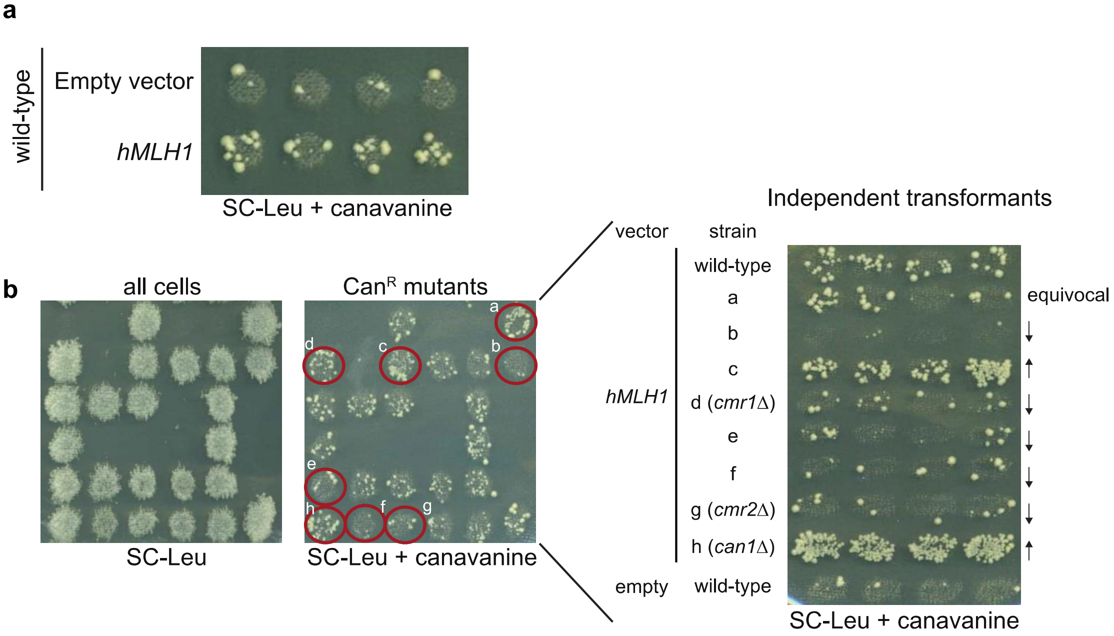

Supplementary Figure 1: Genome-wide identification of factors that influence forward mutation rates. (**a**) Experimental design. Expression of hMLH1 in yeast causes increased mutations in the *CAN1* gene. (**b**) Experimental setup of the genome-wide screen. A collection of 4800 gene deletion strains (EUROSCARF) was transformed with a plasmid expressing hMLH1 (pEH333) (referred to as "all cells") and mutation frequencies were assessed on canavanine containing medium[1]. Mutants with increased or decreased mutation frequencies were re-transformed and four independent transformants assessed for the frequency of canavanine resistance (Can^R, right panel). "Equivocal" hits were eliminated from the analysis. Examples of mutants with increased or decreased mutation rates are reported. From this screen, 667 gene deletions either increased or decreased the inherent mutation rate and among these, two uncharacterized ORFs were named *CMR1* (*YDL156W*, strain d) and *CMR2* (*YOR093C*, strain g). A *can1*Δ mutant (strain h) was used as a negative control.

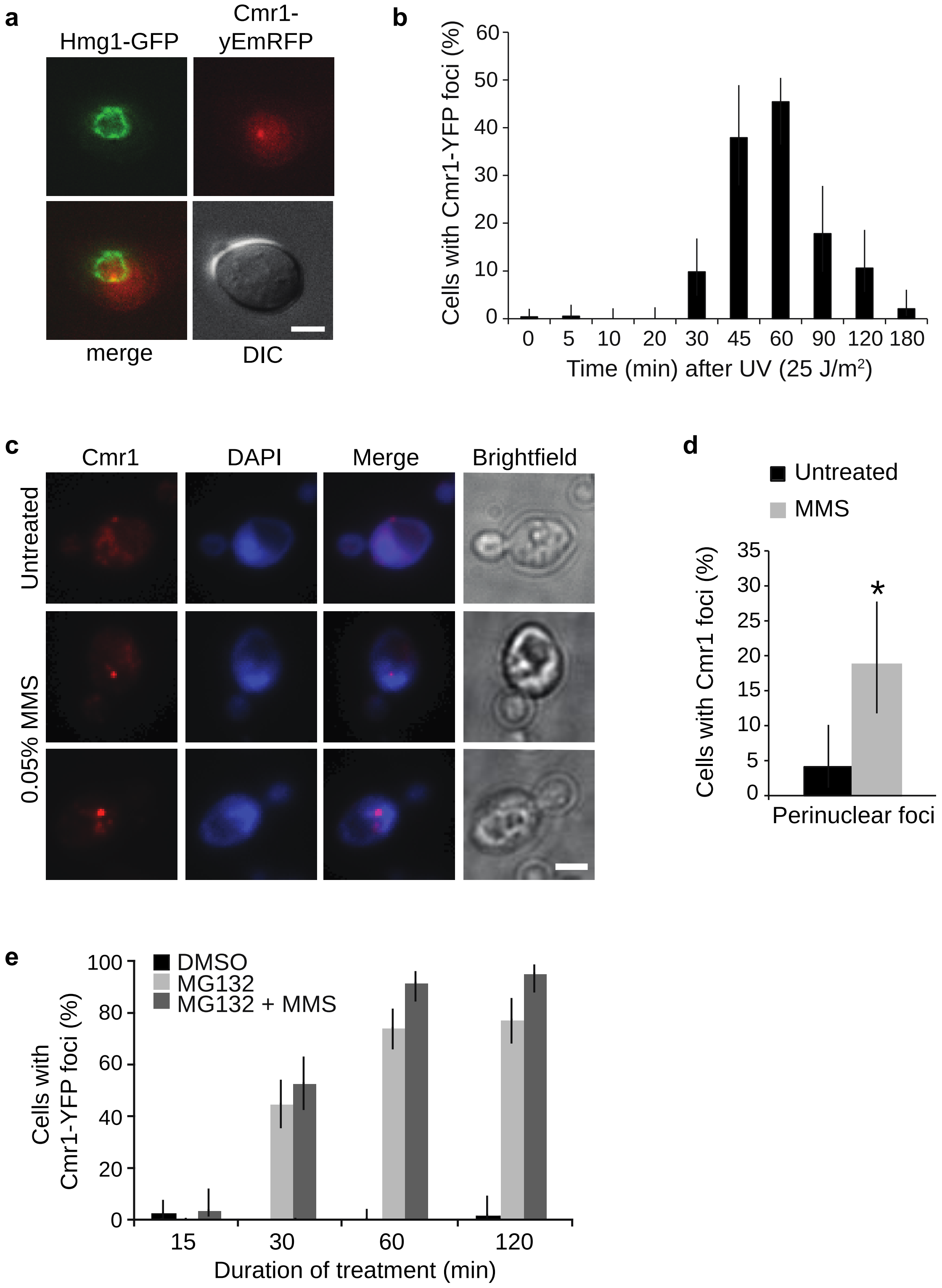

Supplementary Figure 2: Native Cmr1 localizes to perinuclear foci in response to replication stress. (**a**) Cmr1-yEmRFP foci localize inside the nucleus. Nuclear envelope is visualized by Hmg1-GFP in cells treated with 0.05% MMS. Scale bar is equal to 3 µm. IG111 was crossed to Hmg1-GFP expressing strain from the GFP collection and diploids were imaged. (**b**) Kinetics of UV-induced Cmr1 foci. Cmr1-YFP foci were quantified in strain IG66 at different time points after UV-irradiation.

(**c**) Wild-type cells (ML8-9A), untreated or treated with 0.05% MMS for 2 hours were fixed and subjected to immunostaining with an anti-Cmr1 primary antibody, followed by staining with an Alexa Fluor 594 conjugated secondary antibody. DAPI staining was performed before imaging. Scale bar 3 μm. (**d**) Percentage of cells containing Cmr1 foci was quantified. Bright foci localizing in the cytoplasm were defined as background, while perinuclear foci were considered as Cmr1 positive. Error bars represent 95% confidence intervals from two replicates ($n > 150$). *, P-value < 0.02. (**e**) Cmr1 re-localizes into foci upon proteasome inhibition. Cells (IG66) expressing Cmr1-YFP were incubated in the absence or presence of MG132 (75 μg ml^{-1}) or MG132 and MMS (0.05%) for the indicated time, and the percentage of cells with foci was quantified. All incubations were performed in the presence of 3.7% DMSO. Error bars represent 95% confidence intervals. Three replicates ($n > 200$).

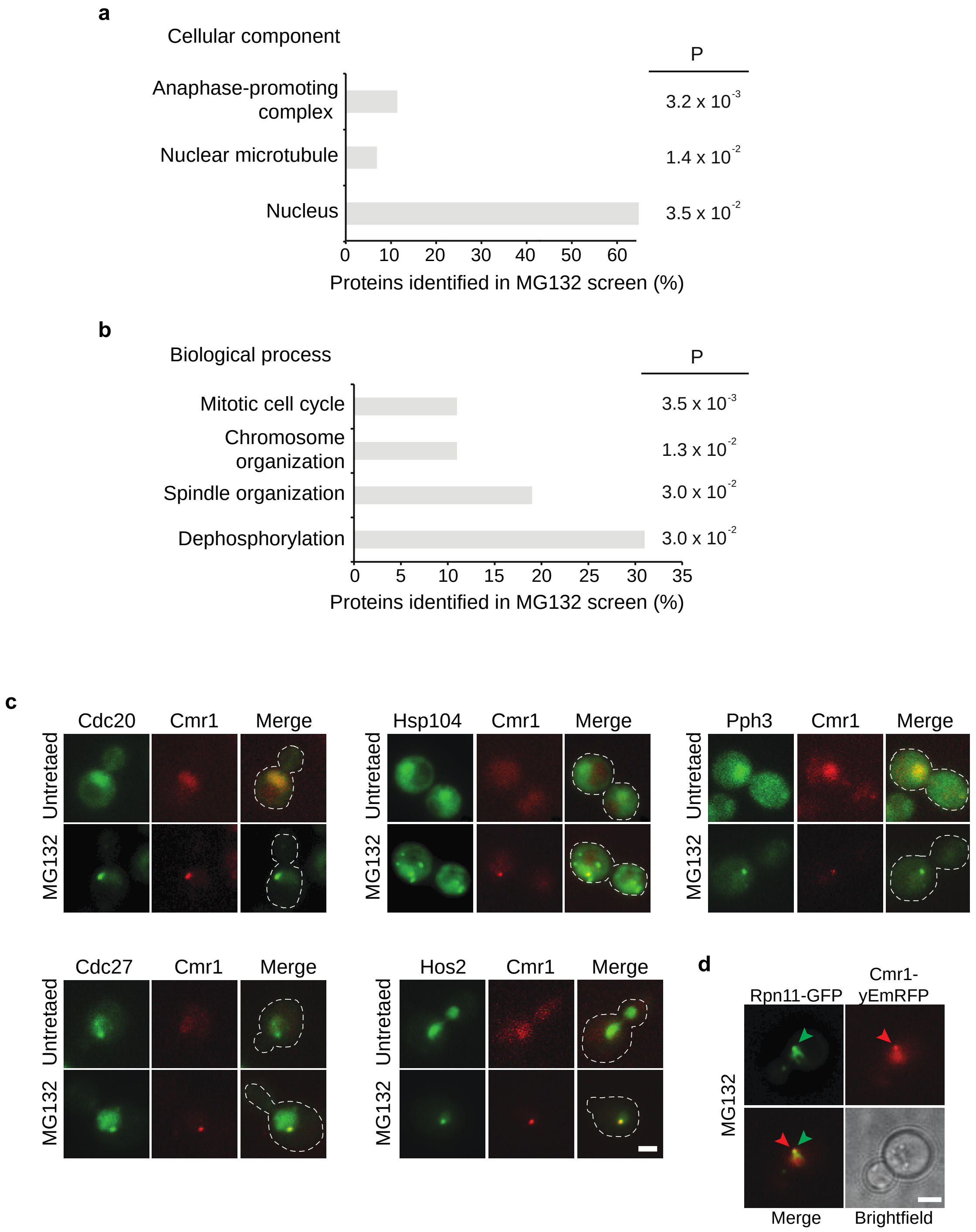

Supplementary Figure 3: Cmr1 co-localizes with proteins from a small subset of GO groups after proteasome inhibition. GO enrichment analysis of Cmr1 co-localizing proteins after MG132 treatment. Significantly over-represented GO cellular components (**a**) or biological processes (**b**) are shown. Bars indicate the percentage of Cmr1 co-localizing proteins belonging to the indicated GO term. The BinGO software[2] was used to calculate the enrichment and the significance of the enrichment was determined by Fisher's test. P-values were corrected using the Benjamini and Hochberg false discovery rate correction. (**c**) Examples of proteins co-localizing with

Cmr1. Cells expressing Cmr1-yEmRFP and the indicated GFP-fusion protein were imaged by fluorescence microscopy before and after treatment with MG132 (75 µg ml^{-1}). Dashed line indicates cell perimeter. Scale bar is equal to 3 µm. (**d**) The proteasome accumulates at INQ. Cells expressing Rpn11-GFP and Cmr1-yEmRFP (IG328-4C) were imaged after MG132 treatment for 1 hour. Arrowheads mark co-localizing foci. Bar equals 3 µm.

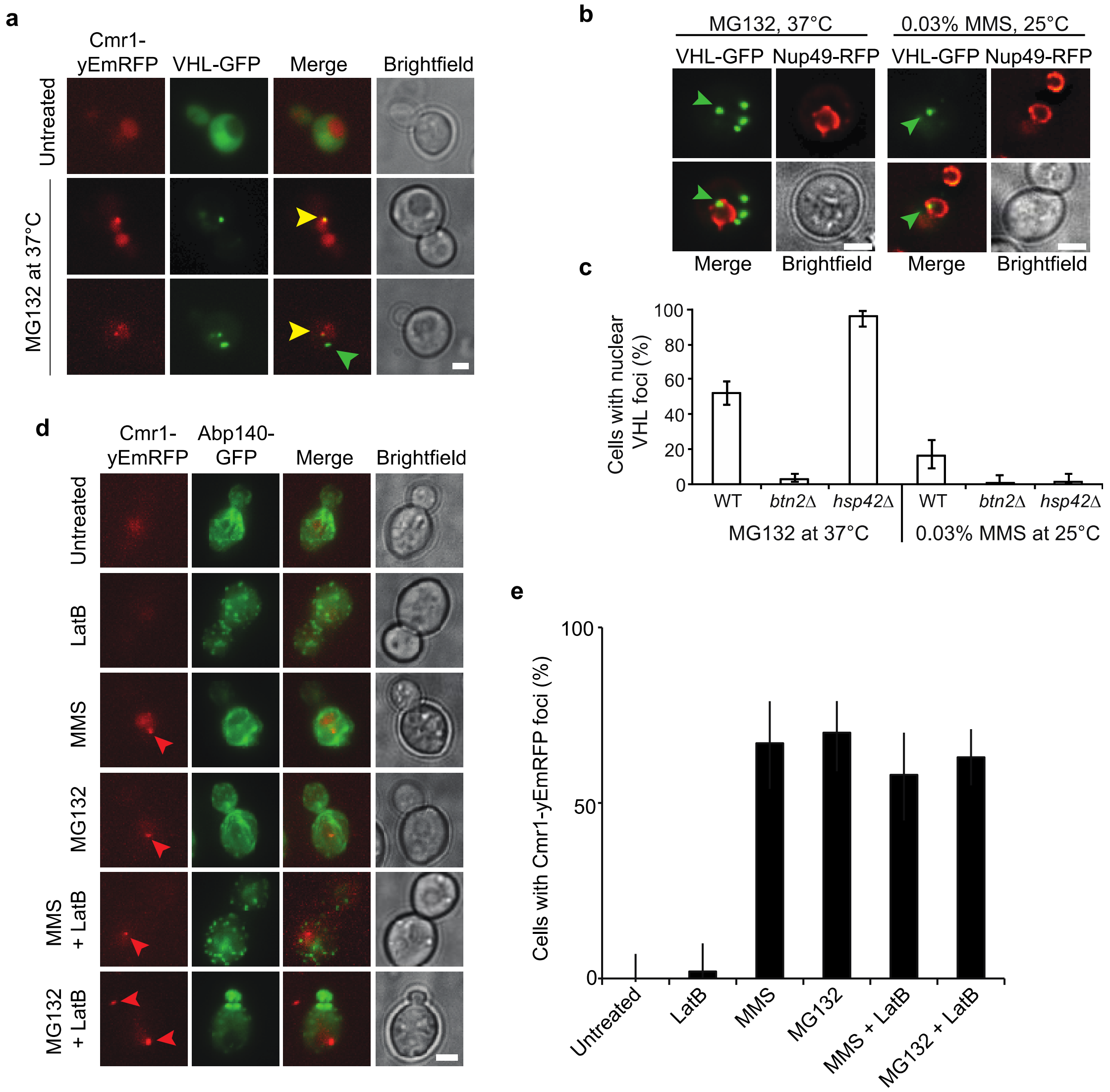

Supplementary Figure 4: Cmr1 re-localization into INQ does not depend on the actin cytoskeleton. (**a**) VHL foci co-localize with Cmr1. Cells expressing Cmr1-yEmRFP (IG111) were transformed with pESC-VHL-GFP and imaged at 25°C and at 37°C with addition of 75 µg ml^{-1} MG132 after galactose induction for 3 hours. Cmr1 colocalizes with VHL at a JUNQ-like perinuclear focus (INQ, yellow arrowhead) but not at the IPOD (green arrowhead). Scale bar 2 µm. (**b**) A proportion of misfolded VHL foci reside in the nucleus. Localization of VHL-GFP (pESC-VHL-GFP) was monitored in wild-type cells (ML8-9A) at 37°C in the presence of MG132 (50 µg ml^{-1}) or at 25°C after treatment with 0.03% MMS for 1 hour. Fifty-two percent of the VHL foci (green arrowheads) were observed inside the nuclear membrane marked by Nup49-yEmRFP (pNEB32). (**c**) Genetic requirements for nuclear foci of misfolded VHL. The experiment in panel **b** was quantified in wild-type (ML8-9A), *btn2*Δ (CC1-2D) and *hsp42*Δ (CC2-2B) strains for two independent experiments (n > 150). Error

bars represent 95% confidence intervals. No cytoplasmic VHL foci were observed after MMS treatment. (**d**) Cmr1 re-localization to foci is not perturbed by inhibition of actin polymerization. Cells expressing Cmr1-yEmRFP and the actin binding protein Abp140-GFP (IG111 crossed to Abp140-GFP strain from GFP collection) untreated or treated with MMS (0.05%) or MG132 (75 µg ml^{-1}) for 2 hours were subjected to simultaneous treatment with latrunculin B (50 µM) to inhibit actin polymerization, and imaged by fluorescence microscopy. Abp140 was used as a marker for actin filaments (first row) or actin patches (second row). Arrowheads indicate Cmr1 foci. Scale bar is equal to 2 µm. (**e**) Quantification of Cmr1 re-localization dependency on the actin cytoskeleton. Percentage of cells in panel **d** exhibiting Cmr1 foci were quantified after treatment with different combinations of drugs. Error bars represent 95% confidence intervals. Two replicates, n > 100.

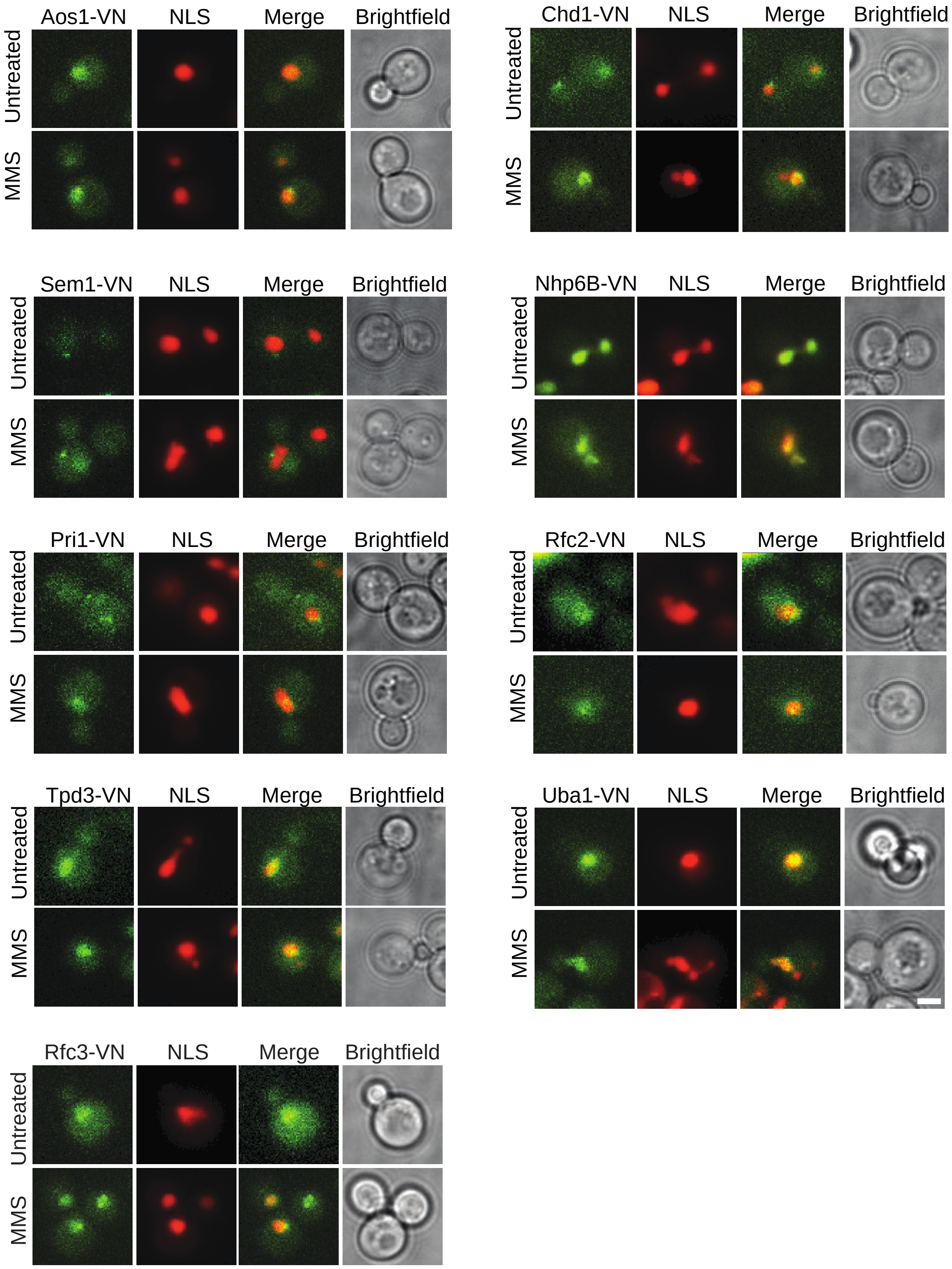

Supplementary Figure 5: Cmr1 interactors from BiFC screen. Examples of VC-VN interaction signals. Strains expressing Cmr1-VC, the indicated VN-fusion protein and NLS-yEmRFP were subjected to fluorescence microscopy before and after treatment with 0.05% MMS. Yellow fluorescent signal represents the VC-VN interaction. Scale bar is equal to 2 µm.

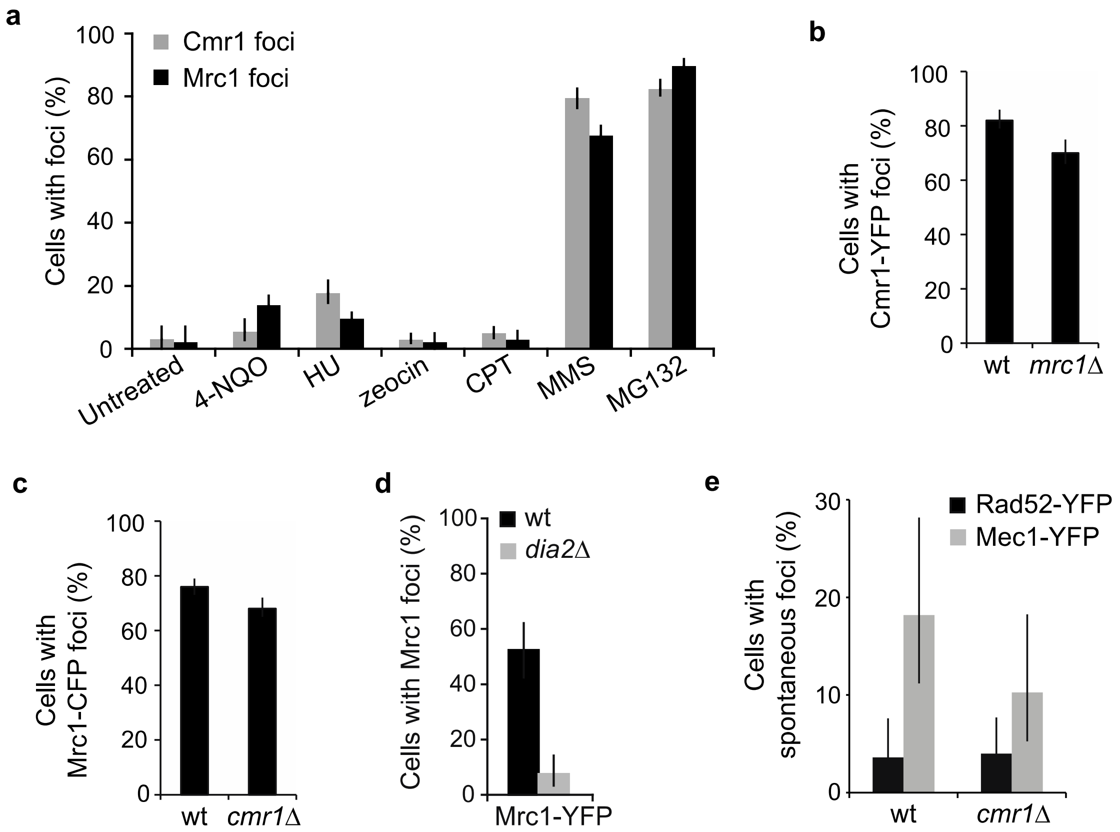

Supplementary Figure 6: Cmr1 and Mrc1 form similar but independent foci. (**a**) Mrc1 and Cmr1 accumulates into nuclear foci in response to replication stress and proteasome inhibition. Endogenously tagged Mrc1-CFP and Cmr1-YFP localization was followed by fluorescence microscopy in haploid strains (IG148 and IG66) treated with DNA damaging agents and in untreated cells. Cells were grown to exponential phase and then treated with zeocin (200 µg ml^{-1}), MMS (0.05%), CPT (5 µg ml^{-1}), 4-NQO (0.2 µg ml^{-1}), HU (200 mM) or MG132 (75 µg ml^{-1}) for 2 hours. Percentage of cells containing foci was quantified. (**b**) Cmr1 foci are independent of Mrc1. Wild-type (IG66) and *mrc1*Δ (IG155-3A) strains expressing Cmr1-YFP were imaged after treatment with 0.05% MMS for 2 hours. (**c**) Mrc1 foci are independent of Cmr1. Wild-type (IG148) and *cmr1*Δ (IG154-7D) strains expressing Mrc1-CFP were imaged after treatment with 0.05% MMS for 2 hours (**d**) Dia2 is required for re-localization of Mrc1 to INQ. Mrc1-YFP focus formation was assessed by fluorescence microscopy in wild-type (IG147) and *dia2*Δ (CC37-5A) cells after treatment with MMS for 2 hours. (e) *cmr1*Δ does not result in increased spontaneous genomic instability. Spontaneous Mec1 and Rad52 foci were quantified in wild-type and *cmr1*Δ cells. Mec1 was tagged endogenously (ML41-4B and ML801-5B), while Rad52-YFP was expressed from the pWJ1213 plasmid (in ML8-9A and DP1). In

panels **a-e**, two-three independent experiments (n > 100) were performed. Error bars represent 95% confidence intervals.

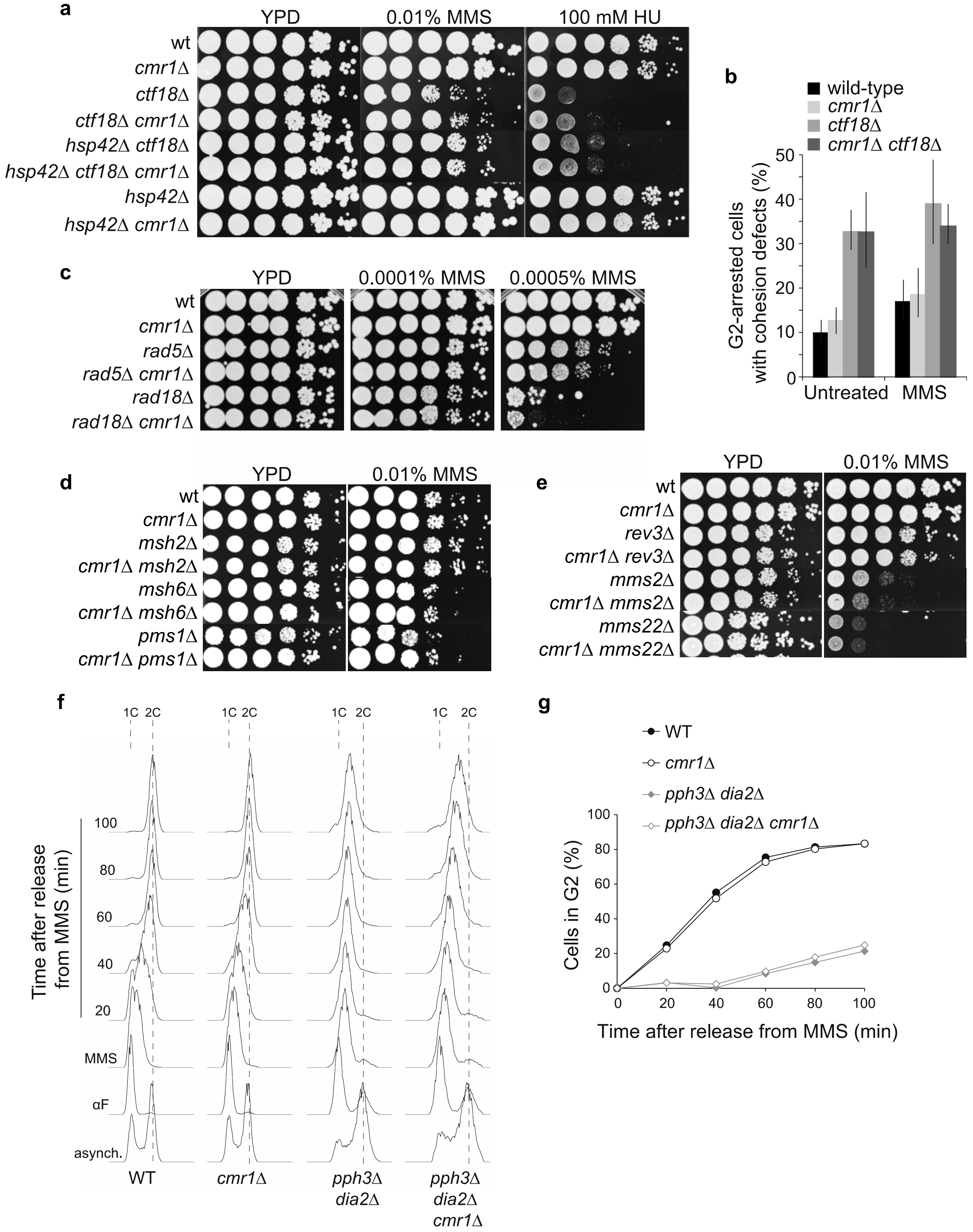

Supplementary Figure 7: *CMR1* epistasis analysis. (**a**) *cmr1*Δ is epistatic with *hsp42*Δ in suppressing MMS and HU sensitivity of the *ctf18*Δ mutant. Ten-fold serial dilutions for each of the indicated genotypes were plated on YPD or YPD containing the indicated drug. Strains were ML8-9A (wt), DP1 (*cmr1*Δ*),* IG177-9C (*ctf18*Δ), IG177-8C (*ctf18*Δ *cmr1*Δ*),* IG292-11B (*hsp42*Δ *ctf18*Δ), CC27-4C (*hsp42*Δ *ctf18*Δ *cmr1*Δ), IG237-18A (*hsp42*Δ) and IG237-17C (*hsp42*Δ *cmr1*Δ). (**b**) Cmr1 does not play a direct role in sister chromatid cohesion. Cells carrying *TetO* tandem repeats on

chromosome *V* and expressing TetR-RFP were arrested in G2 with nocodazole and the percentage of cells with precocious sister chromatid separation (2 RFP dots)[3] was quantified in wild-type (IG310-3B), *cmr1*Δ (IG310-15D), *ctf18*Δ (IG310-20B) and *cmr1*Δ *ctf18*Δ (IG311-1B) strains. At least 300 cells from two replicate experiments were analyzed for each strain. Error bars represent 95% confidence intervals. (**c**-**e**) Epistasis analysis of *cmr1*Δ with DNA repair pathways. Genetic interaction of *cmr1*Δ with mutants in template switching (**c**), mismatch repair (**d**), and postreplication repair and translesion synthesis (**e**). Ten-fold serial dilutions for each of the indicated genotypes were plated on YPD or YPD containing MMS. Strains were ML8-9A (wt), DP1 (*cmr1*Δ*)*, CC25-4A (*rad5*Δ*)*, CC25-1A (*rad5*Δ *cmr1*Δ), IG265-17A (*rad18*Δ), IG265-4D (*rad18*Δ *cmr1*Δ), *msh2*Δ (IG118-6C), *cmr1*Δ *msh2*Δ (IG118-17C), *msh6*Δ (IG119-4D), *cmr1*Δ *msh6*Δ (IG119-8D), *pms1*Δ (IG120-6B), *cmr1*Δ *pms1*Δ (IG120-2B), *rev3*Δ (IG190-1C), *cmr1*Δ *rev3*Δ (IG190-4D), *mms2*Δ (IG267-20D), *cmr1*Δ *mms2*Δ (IG267-9A), *mms22*Δ (IG260-2D), *cmr1*Δ *mms22*Δ (IG260-1D). (**f**) *cmr1*Δ mutant cells are proficient for replication restart after MMS treatment. Wild-type (CC44-8A), *cmr1*Δ (DP1), *dia2*Δ *pph3*Δ (IG296-2C), and *dia2*Δ *pph3*Δ *cmr1*Δ (IG296-49B) strains arrested in G1 with α-factor were released into YPD containing 0.03% MMS for 45 min followed by release into YPD containing 15 μg ml^{-1} nocodazole. Samples were harvested and stained with propidium iodide for flow cytometry analysis at the indicated time points. Representative profiles are shown. **(g)** Quantification of the percentage of cells in G2 phase at the individual time points following MMS-release in **f**. Graph shows average of 2-4 independent experiments.

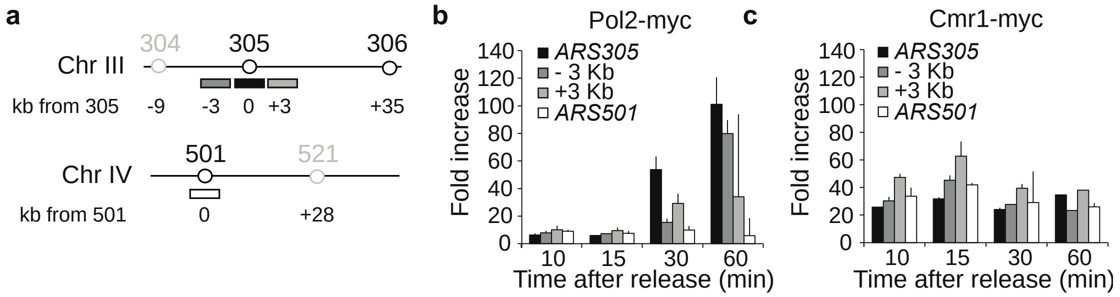

Supplementary Figure 8: Cmr1 constitutively associates with chromatin. (**a**) Schematic representation of genomic regions containing the early-firing origin ARS305 and late-firing origin ARS501. Boxes indicate qPCR probes location at ARS305 (black), -3 kb and + 3 kb from ARS305 (grey) and at ARS501 (white). Inactive ARS are depicted in grey and distances from the ARS of interest are reported. (**b-c**) ChIP experiments were performed on myc-tagged Cmr1 (IG309) and Pol2 (LBy49) cells. G1-arrested cells were released in 0.2 M HU for the indicated times. The height of the bars represents the real-time PCR signal as fold increase of the IP over the beads-only control for Pol2 (**b**) and Cmr1 (**c**). Error bars represent the standard deviation of two to four replicates.

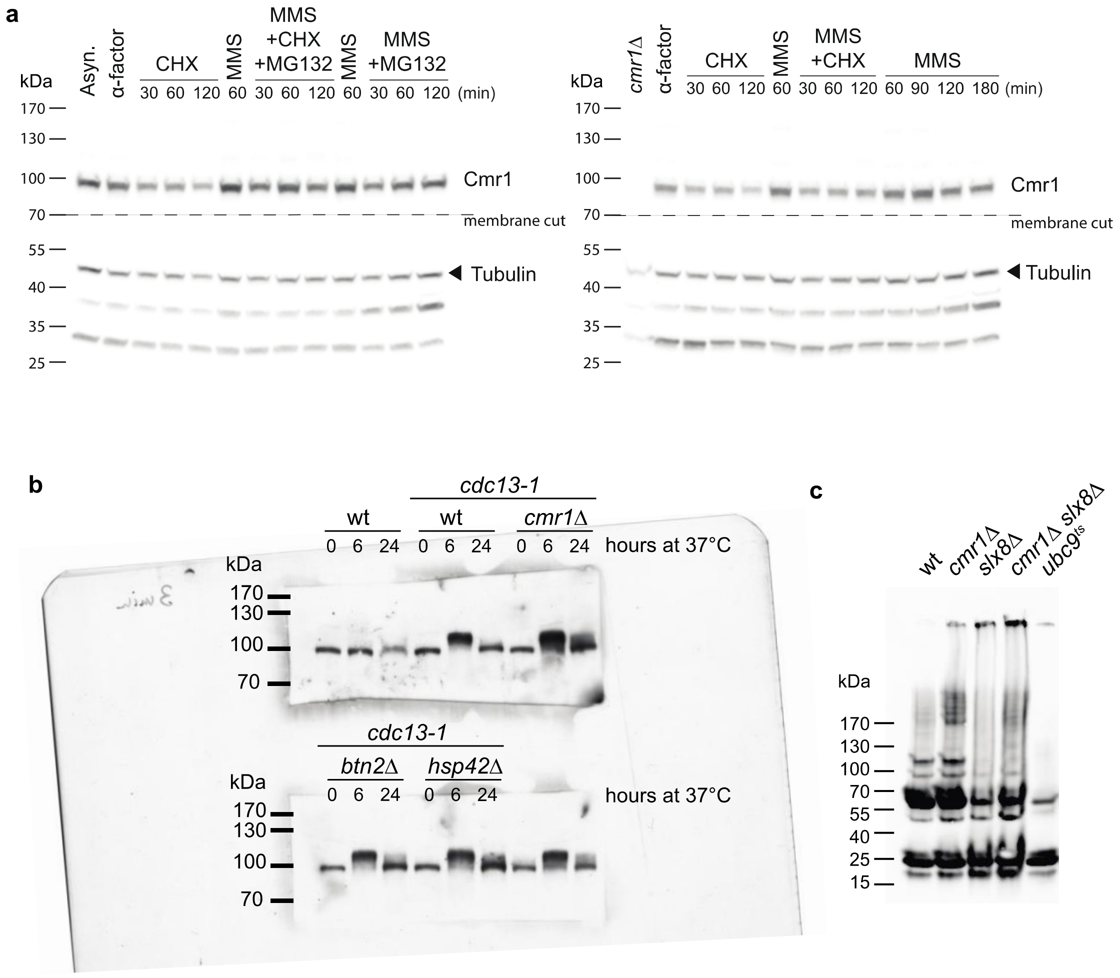

Supplementary Figure 9: Uncropped immunoblots. (**a**) Uncropped immunoblot of Fig. 3g. (**b**) Uncropped immunoblot of Fig. 6c. (**c**) Uncropped immunoblot of Fig. 6f.

Supplementary tables:

Supplementary Table 1 Unique Cmr1 interactions from BiFC screen. Hits in bold have been manually confirmed.

Adk1	**Gim5**	Lsm4	**Rfc3**	Stf2	Ymr122w-A
**Aos1**	Gir2	**Mcm3**	**Rnr4**	Sto1	Ylr296w
Aro4	Gmp1	Med8	**Rod1**	Tfb4	Ymr221c
**Bmh1**	Grx1	Mtf1	Rpb3	Tma17	Ynl134c
Cdc60	Grx8	Mti1	Rpb8	**Tpd3**	Ypl245w
**Chd1**	Gsp2	**Nhp6B**	Rpc10	Tpo1	Ypr1
Cpa2	**Hsc82**	**Nup133**	Rpp2A	**Uba1**	Ypt31
Cpr6	**Hsp26**	Nut2	Rri2	Uga1	Ypt32
Crs5	Hxk1	Osw1	Rsb1	Utp10	Zuo1
Cyc2	Hxk2	Pbi2	**Sem1**	Ybr085c-A	
Dot5	Idi1	Pml39	**Sgf11**	Ydr387c	
Egd2	Ils1	**Pri1**	Smx3	Yel047c	
Gad1	Ipp1	Ras2	**Snf6**	Yjl216c	
Gdi1	**Ixr1**	**Rfc2**	Sod1	Ylr363w-A	

Supplementary Table 2 Manually curated *CMR1* genetic interactions in replication stress. -/+, 10-fold. - -/++, 100-fold. Strains are listed in Supplementary Table 5.

**Positive interactions**		
Mutation	Functions	Strength of interaction
pph3Δ	S-phase replication	+
mrc1Δ	checkpoint and sister	+
ctf18Δ	chromatid cohesion	++
scc1-73		+
mrc1AQ	Replication checkpoint	++
mlp1Δ mlp2Δ	Myosin-like proteins	+
**Negative interactions**		
Mutation	Functions	Strength of interaction
rad52Δ	Homologous	- -
mre11Δ	recombination	- -
rad9Δ	DNA damage checkpoint	-
rad18Δ	E3-ligase-dependent	-
slx8Δ	proteasomal degradation	- -
dia2Δ	and SUMO-dependent	-
hel2Δ	proteasomal degradation	-
**No interaction (epistatic)**		
Mutation	Functions	
csm3Δ	Replication fork stability	
tof1Δ		
dcc1Δ		
elg1Δ		
rad24Δ	Checkpoint	
hta1-S129A hta2-S129A		
msh2Δ	Mismatch repair	
msh6Δ		
msh3Δ		
pms1Δ		
exo1Δ		
rad59Δ	Regulation of homologous	
shu1Δ	recombination	
srs2Δ		
hdf1Δ	Non-homologous end joining	
rad27Δ	DNA replication	
rad10Δ	NER	
rad14Δ		
rev3Δ	TLS	
rad5Δ	Template switching	
apn1Δ	BER	
mms21-11	Post-replication repair	
mms22Δ		
mms2Δ		
ubp10Δ	Ubiquitin-dependent	

*ubc4*Δ	degradation
*btn2*Δ<br>*hsp42*Δ	Unfolded protein response

Supplementary Table 3 *CMR1* genetic interactions from SGA screen. Positive and negative *cmr1*Δ genetic interactions in presence of HU are listed. Hits are ranked by normalized ratio between the growth of comparer/experimental. Cut-off is set at 0.25 for the positive genetic interactions and at 1.50 for the negative genetic interactions.

Positive genetic interactions

ID Column	Gene	P-Value	Z-Score	Normalized Growth Ratio (Comparer:: Exp)	Growth Ratio (Comparer / Exp)
YNL052W	COX5A	7.08E-07	-4.95912	0.10::1.36	0.07
YEL024W	RIP1	1.70E-06	-4.78666	0.10::1.24	0.08
YPL216W	-	1.06E-06	-4.87924	0.10::1.30	0.08
YNL116W	DMA2	1.06E-06	-4.87983	0.10::1.30	0.08
YBR066C	NRG2	2.62E-06	-4.6986	0.10::1.18	0.08
YML027W	YOX1	1.04E-06	-4.88446	0.10::1.31	0.08
YPL201C	YIG1	4.33E-06	-4.5947	0.10::1.12	0.09
YNL032W	SIW14	7.06E-06	-4.49185	0.10::1.06	0.09
YIL103W	DPH1	4.76E-06	-4.57525	0.10::1.11	0.09
YNR027W	BUD17	6.94E-06	-4.49545	0.10::1.06	0.09
YBR125C	PTC4	4.65E-06	-4.57984	0.10::1.11	0.09
YBR288C	APM3	5.43E-06	-4.54747	0.10::1.09	0.09
YDR443C	SSN2	2.74E-06	-4.68911	0.10::1.18	0.09
YLR237W	THI7	2.91E-06	-4.67729	0.10::1.17	0.09
YHR132W-A	IGO2	6.17E-06	-4.52045	0.10::1.07	0.09
YLR390W-A	CCW14	3.62E-06	-4.63219	0.10::1.14	0.09
YBR180W	DTR1	1.41E-05	-4.3422	0.10::0.98	0.1
YIL113W	SDP1	1.52E-05	-4.32612	0.10::0.97	0.1
YNL056W	OCA2	9.07E-06	-4.43823	0.10::1.03	0.1
YBR028C	YPK3	1.46E-05	-4.33399	0.10::0.97	0.1
YNL040W	-	1.09E-05	-4.3995	0.10::1.01	0.1
YBR020W	GAL1	1.18E-05	-4.38047	0.10::1.00	0.1
YNL008C	ASI3	8.65E-06	-4.4485	0.10::1.03	0.1
YNL046W	-	1.65E-05	-4.30765	0.10::0.96	0.1
YIR002C	MPH1	1.59E-05	-4.31559	0.10::0.96	0.1
YNL122C	-	1.32E-05	-4.35737	0.10::0.98	0.1
YIL110W	HPM1	1.13E-05	-4.39013	0.15::1.49	0.1
YDL089W	NUR1	1.01E-05	-4.41433	0.10::1.01	0.1

YBR129C	OPY1	1.60E-05	-4.31377	0.10::0.96	0.1
YBL102W	SFT2	1.86E-05	-4.28094	0.10::0.94	0.11
YBR052C	RFS1	2.39E-05	-4.22496	0.10::0.92	0.11
YNL123W	NMA111	2.99E-05	-4.17438	0.10::0.89	0.11
YDR496C	PUF6	2.34E-05	-4.22996	0.10::0.92	0.11
YDL019C	OSH2	3.03E-05	-4.17138	0.10::0.89	0.11
YDR491C	-	3.25E-05	-4.15523	0.10::0.88	0.11
YJL161W	FMP33	4.26E-05	-4.09287	0.10::0.85	0.12
YDL026W	-	5.01E-05	-4.05526	0.10::0.84	0.12
YBR165W	UBS1	6.78E-05	-3.98373	0.10::0.81	0.12
YDR488C	PAC11	3.88E-05	-4.11478	0.10::0.86	0.12
YBL096C	-	9.00E-05	-3.91616	0.15::1.15	0.13
YBR019C	GAL10	7.34E-05	-3.96511	0.10::0.80	0.13
YIL170W	-	8.20E-05	-3.9385	0.15::1.20	0.13
YKR095W	MLP1	7.63E-05	-3.95565	0.10::0.79	0.13
YGR089W	NNF2	0.00045682	-3.50487	0.10::0.62	0.16
YBR130C	SHE3	0.00081044	-3.3492	0.27::1.55	0.17
YDL036C	PUS9	0.00078108	-3.35942	0.10::0.58	0.17
YJL016W	-	0.00093962	-3.30801	0.24::1.34	0.18
YNL139C	THO2	0.0010305	-3.28206	0.10::0.55	0.18
YML121W	GTR1	0.00151124	-3.17252	0.31::1.59	0.19
YKR010C	TOF2	0.00153288	-3.16838	0.10::0.52	0.19
YBR073W	RDH54	0.00138644	-3.19746	0.10::0.53	0.19
YAR002C-A	ERP1	0.0016675	-3.14383	0.22::1.12	0.19
YBR010W	HHT1	0.00168184	-3.14133	0.10::0.51	0.19
YNL273W	TOF1	0.0022438	-3.0559	0.10::0.49	0.2
YJL206C	-	0.0021178	-3.0732	0.10::0.49	0.2
YHR168W	MTG2	0.00296	-2.97186	0.14::0.66	0.21
YKR096W	ESL2	0.0025194	-3.02101	0.29::1.42	0.21
YDL039C	PRM7	0.0025504	-3.01731	0.28::1.37	0.21
YBR043C	QDR3	0.0039566	-2.88161	0.12::0.55	0.22
YDL093W	PMT5	0.0034564	-2.92394	0.26::1.17	0.22
YMR191W	SPG5	0.0032284	-2.94511	0.24::1.11	0.22
YDR474C	-	0.0039456	-2.88248	0.10::0.45	0.22
YIL076W	SEC28	0.0048864	-2.81443	0.18::0.76	0.23
YIL112W	HOS4	0.0045192	-2.83944	0.31::1.37	0.23
YIL152W	-	0.0042596	-2.85827	0.10::0.44	0.23

Negative genetic interactions

ID Column	Gene	P-Value	Z-Score	Normalized Growth Ratio (Comparer:: Exp)	Growth Ratio (Comparer / Exp)
YGR168C	-	1.19E-07	5.29509	1.79::0.10	17.94
YER186C	-	3.42E-07	5.09875	1.61::0.10	16.15
YNL107W	YAF9	6.10E-07	4.98818	1.52::0.10	15.22
YPL212C	PUS1	6.33E-07	4.98094	1.52::0.10	15.16
YGL124C	MON1	7.00E-07	4.96137	1.50::0.10	15

YPL208W	RKM1	7.81E-07	4.94013	1.48::0.10	14.83
YDL149W	ATG9	1.36E-06	4.83047	1.40::0.10	13.98
YER011W	TIR1	2.05E-06	4.74883	1.34::0.10	13.38
YDR410C	STE14	2.10E-06	4.74324	1.33::0.10	13.34
YDL041W	-	2.13E-06	4.7404	1.33::0.10	13.32
YKR035C	OPI8	2.48E-06	4.71002	1.31::0.10	13.11
YPR120C	CLB5	3.36E-06	4.64772	1.27::0.10	12.68
YDR074W	TPS2	4.48E-06	4.5877	1.23::0.10	12.28
YBR127C	VMA2	5.95E-06	4.52809	1.19::0.10	11.89
YPR064W	-	7.31E-06	4.48446	1.16::0.10	11.62
YGR063C	SPT4	7.79E-06	4.47098	1.15::0.10	11.53
YBR084C-A	RPL19A	8.73E-06	4.44634	1.14::0.10	11.38
YLR435W	TSR2	9.53E-06	4.42761	1.19::0.11	11.27
YGR159C	NSR1	9.68E-06	4.42428	1.12::0.10	11.25
YMR198W	CIK1	1.05E-05	4.4061	1.11::0.10	11.14
YBL100C	-	1.16E-05	4.38495	1.10::0.10	11.01
YIL121W	QDR2	1.16E-05	4.38495	1.10::0.10	11.01
YJL200C	ACO2	1.22E-05	4.37377	1.09::0.10	10.95
YDR484W	VPS52	1.24E-05	4.37069	1.09::0.10	10.93
YPL268W	PLC1	1.24E-05	4.37108	1.09::0.10	10.93
YOR270C	VPH1	1.51E-05	4.32775	1.07::0.10	10.68
YNL315C	ATP11	1.67E-05	4.30475	1.05::0.10	10.55
YPL180W	TCO89	1.78E-05	4.2914	1.05::0.10	10.47
YIL116W	HIS5	1.83E-05	4.2842	1.04::0.10	10.43
YBL099W	ATP1	1.85E-05	4.28185	1.04::0.10	10.42
YDL157C	-	2.15E-05	4.24867	1.02::0.10	10.24
YBR189W	RPS9B	2.45E-05	4.21957	1.01::0.10	10.08
YKR019C	IRS4	2.45E-05	4.21957	1.01::0.10	10.08
YDL090C	RAM1	2.53E-05	4.21221	1.00::0.10	10.04
YHL005C	-	2.59E-05	4.20655	1.00::0.10	10.01
YGR056W	RSC1	2.78E-05	4.19095	0.99::0.10	9.93
YDL020C	RPN4	2.96E-05	4.17661	0.98::0.10	9.85
YHR005C	GPA1	3.26E-05	4.15416	0.97::0.10	9.73
YDR048C	-	3.31E-05	4.15136	0.97::0.10	9.72
YDR234W	LYS4	3.55E-05	4.13515	0.96::0.10	9.63
YLR192C	HCR1	3.63E-05	4.13017	0.96::0.10	9.61
YNL041C	COG6	3.84E-05	4.11694	0.95::0.10	9.54
YDL042C	SIR2	3.92E-05	4.11229	0.95::0.10	9.52
YNR037C	RSM19	4.57E-05	4.07662	0.93::0.10	9.34
YPR139C	LOA1	5.41E-05	4.0372	0.91::0.10	9.14
YKL204W	EAP1	6.41E-05	3.99707	1.44::0.16	8.95
YNL138W	SRV2	6.45E-05	3.99581	0.89::0.10	8.94
YJL101C	GSH1	8.23E-05	3.93755	0.87::0.10	8.66
YLR358C	-	8.28E-05	3.93614	0.87::0.10	8.66
YIR009W	MSL1	8.48E-05	3.93035	0.86::0.10	8.63
YBR058C	UBP14	8.58E-05	3.92751	0.86::0.10	8.62
YJL175W	-	9.78E-05	3.89591	0.85::0.10	8.47
YBR081C	SPT7	0.00011076	3.86573	0.83::0.10	8.34
YDL156W	CMR1	0.00011610	3.8542	0.83::0.10	8.29
YOL004W	SIN3	0.00014089	3.80659	0.81::0.10	8.08

YNR052C	POP2	0.00014549	3.79864	0.80::0.10	8.04
YGL107C	RMD9	0.00015094	3.78951	0.80::0.10	8
YCL037C	SRO9	0.00017027	3.75947	0.79::0.10	7.88
YBR163W	EXO5	0.00019463	3.72589	0.77::0.10	7.74
YIL154C	IMP2'	0.00020706	3.71025	0.77::0.10	7.67
YGL033W	HOP2	0.00022264	3.69183	0.76::0.10	7.6
YDR497C	ITR1	0.00023218	3.68116	0.76::0.10	7.55
YBL006C	LDB7	0.0002503	3.66196	0.75::0.10	7.48
YDR512C	EMI1	0.0002538	3.65839	0.75::0.10	7.46
YLR337C	VRP1	0.00027766	3.63531	0.74::0.10	7.37
YDR266C	HEL2	0.00028102	3.6322	0.74::0.10	7.36
YNR010W	CSE2	0.0002796	3.63349	0.74::0.10	7.36
YLR021W	IRC25	0.00035296	3.57297	0.71::0.10	7.13
YGR155W	CYS4	0.00043012	3.52088	0.69::0.10	6.93
YJR055W	HIT1	0.0004502	3.50876	1.11::0.16	6.89
YKL139W	CTK1	0.0004829	3.49007	1.11::0.16	6.82
YDR159W	SAC3	0.00049082	3.48572	0.68::0.10	6.8
YNL073W	MSK1	0.00057682	3.44229	0.66::0.10	6.64
YLR382C	NAM2	0.00060316	3.43019	1.11::0.17	6.6
YPR087W	VPS69	0.00077534	3.36145	0.64::0.10	6.36
YJL151C	SNA3	0.000797	3.35383	1.10::0.17	6.34
YBR131W	CCZ1	0.00089938	3.32025	1.60::0.26	6.22
YLR421C	RPN13	0.00098774	3.29399	1.14::0.19	6.14
YNR006W	VPS27	0.00109812	3.2641	0.60::0.10	6.04
YLR404W	FLD1	0.0011177	3.25909	1.33::0.22	6.02
YPR024W	YME1	0.00123894	3.22976	0.59::0.10	5.93
YMR116C	ASC1	0.0017358	3.13207	0.56::0.10	5.63
YLR025W	SNF7	0.0017455	3.13043	0.56::0.10	5.62
YPL181W	CTI6	0.0020524	3.08255	0.76::0.14	5.48
YBR082C	UBC4	0.0023808	3.0381	0.99::0.18	5.35
YKL080W	VMA5	0.0024226	3.03284	0.53::0.10	5.34
YDR463W	STP1	0.0024182	3.0334	1.07::0.20	5.34
YOR058C	ASE1	0.0029132	2.97676	0.52::0.10	5.18
YML071C	COG8	0.0030496	2.96269	0.51::0.10	5.14
YLR320W	MMS22	0.0034974	2.92026	0.50::0.10	5.02
YOR141C	ARP8	0.003995	2.87856	0.49::0.10	4.91
YGR064W	-	0.0044256	2.84611	0.48::0.10	4.83
YML014W	TRM9	0.00463	2.83171	0.48::0.10	4.79
YAL035W	FUN12	0.0048182	2.81895	0.48::0.10	4.76
YMR125W	STO1	0.005162	2.79674	0.47::0.10	4.7
YLR193C	UPS1	0.005377	2.78354	0.47::0.10	4.67
YNL252C	MRPL17	0.0055106	2.77556	0.66::0.14	4.65
YHL033C	RPL8A	0.005772	2.76046	0.46::0.10	4.61
YBR030W	RKM3	0.006388	2.72717	0.85::0.19	4.53
YNL292W	PUS4	0.006571	2.71784	0.71::0.16	4.51
YOR147W	MDM32	0.007423	2.67724	0.62::0.14	4.41
YFR025C	HIS2	0.0074706	2.67511	0.44::0.10	4.4
YDL045W-A	MRP10	0.0083628	2.63706	0.97::0.23	4.32
YOR096W	RPS7A	0.0084088	2.6352	0.49::0.11	4.31
YHR012W	VPS29	0.0088146	2.61916	0.43::0.10	4.27

YGL243W	TAD1	0.0106718	2.55327	1.85::0.45	4.13
YGL219C	MDM34	0.0119464	2.51373	0.40::0.10	4.04
YDR431W	-	0.0125	2.49771	0.65::0.16	4
YDR456W	NHX1	0.012712	2.49174	0.40::0.10	3.99
YNL021W	HDA1	0.0131938	2.4785	1.06::0.27	3.96
YBR045C	GIP1	0.0136422	2.46655	1.09::0.28	3.94
YAL013W	DEP1	0.0140212	2.45672	0.39::0.10	3.92
YGR204W	ADE3	0.0146034	2.44207	0.39::0.10	3.89
YLR228C	ECM22	0.0157458	2.41475	1.50::0.39	3.83
YDR076W	RAD55	0.0173694	2.37879	0.41::0.11	3.76
YMR048W	CSM3	0.0176264	2.37337	1.19::0.32	3.75
YNR036C	MRPS12	0.021604	2.29726	1.08::0.30	3.6
YLR405W	DUS4	0.0218	2.29383	1.37::0.38	3.59
YPL055C	LGE1	0.021692	2.29573	0.36::0.10	3.59
YJL160C	-	0.022234	2.28634	0.93::0.26	3.58
YNL315C	ATP11	0.025482	2.23402	0.35::0.10	3.48
YNL051W	COG5	0.027548	2.20365	1.14::0.33	3.42
YNL111C	CYB5	0.027544	2.20371	0.94::0.28	3.42
YBR016W	-	0.028172	2.19488	1.39::0.41	3.4
YLR110C	CCW12	0.028714	2.18739	1.04::0.31	3.39
YNL250W	RAD50	0.029366	2.17853	0.53::0.16	3.38
YGL258W	VEL1	0.03056	2.16277	1.10::0.33	3.35
YDL069C	CBS1	0.03793	2.07562	1.20::0.38	3.19
YJL159W	HSP150	0.039596	2.05795	1.04::0.33	3.16
YML041C	VPS71	0.039594	2.05796	0.92::0.29	3.16
YPL182C	-	0.042964	2.02406	0.31::0.10	3.11
YIL166C	-	0.042912	2.02457	1.04::0.33	3.11
YDR506C	GMC1	0.044696	2.0075	1.06::0.34	3.08
YKL081W	TEF4	0.045414	2.0008	0.33::0.11	3.07
YGL257C	MNT2	0.04544	2.00056	1.67::0.54	3.07
YDL006W	PTC1	0.045398	2.00095	1.00::0.33	3.07
YBR121C	GRS1	0.045962	1.99575	1.20::0.39	3.06
YLR006C	SSK1	0.05075	1.95359	0.38::0.13	2.99
YHL020C	OPI1	0.05207	1.94256	1.21::0.41	2.97
YIL052C	RPL34B	0.052492	1.93908	0.30::0.10	2.97
YPL144W	POC4	0.05444	1.92332	0.29::0.10	2.94
YDR181C	SAS4	0.055594	1.9142	1.88::0.64	2.93
YPL065W	VPS28	0.056928	1.90386	0.53::0.18	2.91
YPL101W	ELP4	0.05766	1.89827	1.09::0.37	2.9
YBR231C	SWC5	0.059258	1.88628	0.29::0.10	2.89
YGL064C	MRH4	0.059724	1.88283	0.39::0.14	2.88
YBR095C	RXT2	0.063216	1.85767	1.32::0.46	2.84
YDR432W	NPL3	0.064902	1.84594	0.28::0.10	2.82
YHR033W	-	0.066062	1.838	0.82::0.29	2.81
YKL040C	NFU1	0.067326	1.82949	0.34::0.12	2.8
YGL195W	GCN1	0.069452	1.81547	0.71::0.25	2.78
YNL022C	-	0.070368	1.80954	0.93::0.33	2.77
YER137C	-	0.071104	1.80481	0.98::0.36	2.76
YER155C	BEM2	0.071802	1.80037	0.83::0.30	2.76
YFL036W	RPO41	0.072226	1.79769	0.28::0.10	2.75

YER103W	SSA4	0.072048	1.79881	0.99::0.36	2.75
YNL294C	RIM21	0.07406	1.78625	0.27::0.10	2.74
YCR008W	SAT4	0.074908	1.78103	0.60::0.22	2.73
YDL013W	SLX5	0.075142	1.77959	1.11::0.41	2.73
YLL033W	IRC19	0.076726	1.77001	0.27::0.10	2.71
YJL128C	PBS2	0.076868	1.76916	0.97::0.36	2.71
YHR028C	DAP2	0.081784	1.74043	1.00::0.37	2.67
YNL284C	MRPL10	0.085468	1.7198	0.72::0.27	2.64
YPL106C	SSE1	0.086288	1.71531	0.85::0.32	2.63
YIL134W	FLX1	0.087476	1.70887	1.01::0.39	2.62
YBR151W	APD1	0.090564	1.69243	1.01::0.39	2.6
YPR189W	SKI3	0.091872	1.68561	0.44::0.17	2.59
YKR085C	MRPL20	0.0984	1.65266	0.55::0.22	2.55
YMR003W	AIM34	0.100316	1.64332	0.41::0.16	2.53
YJL136C	RPS21B	0.100308	1.64336	0.49::0.19	2.53
YNR020C	ATP23	0.102062	1.63494	1.23::0.49	2.52
YPL183W	-	0.102178	1.63438	0.61::0.24	2.52
YOR030W	DFG16	0.103264	1.62923	0.35::0.14	2.51
YHR206W	SKN7	0.104136	1.62512	0.25::0.10	2.51
YER119C	AVT6	0.109278	1.60144	1.10::0.45	2.48
YOR082C	-	0.116478	1.56973	1.29::0.53	2.44
YBR098W	MMS4	0.118116	1.56273	0.97::0.40	2.43

Supplementary Table 4 WDR76 physical interactions. Identified proteins with above 1.6-fold difference in both H/L and L/H normalized ratios are listed. Hits are ranked by normalized H/L ratio.

Protein ID	Unique peptides	Unique sequence coverage (%)	HeLa H/L (reverse)	HeLa L/H (forward)
WDR76	16	51.3	100.21	31.40
HELLS	8	24.3	10.25	8.02
HSPC075	7	3.9	6.13	3.70
CCT2	8	50.7	5.35	2.26
XRCC6	13	37.8	5.27	3.05
XRCC5	13	31.3	5.20	2.99
ZC3HAV1	4	22.7	5.01	2.95
HLC7	2	24	4.75	1.78
SUGT1	2	25.8	4.09	1.78
LAMBR	7	32.3	3.83	1.74
HSP90A	9	15.2	3.56	1.68
ADPRT	27	38.3	3.49	3.03
BAG2	3	31.3	3.00	1.91
CCT7	9	46.2	2.93	1.66
CCT8	14	48.9	2.84	1.87
CCT5	13	45.5	2.81	1.71
CCT3	13	41.7	2.64	1.74
CYPB	3	40.7	2.48	4.92
LRRC59	3	11.4	2.38	3.54
CCT4	13	53.1	2.36	1.60
CFIM25	3	36.6	2.30	7.15
FXR1	13	33.1	2.08	1.60
C1QBP	2	21.1	2.06	1.52
HMGB1	2	19	2.01	2.09
RPS23	4	30.1	1.75	1.70
RPL22	3	30.5	1.74	2.40
FACT80	5	17.6	1.72	1.71
RPS18	10	58.6	1.71	2.00
RPS25	4	24	1.64	2.69
FEN1	7	27.9	1.61	1.65
RPS17	2	15.4	1.59	3.72

Supplementary Table 5 Yeast strains used in this study. Yeast strains in this study are derivatives of ML8-9A, a *RAD5 ADE2* derivative of W303-1A (*MAT***a** *BAR1 LYS2 ade2-1 can1-100 ura3-1 his3-11,15 leu2-3, 112 trp1-1 rad5-535*)[4].

Strain	Genotype	Source
CC1-3B	*MAT***a** *btn2::KanMX MRC1-4ala-YFP*	This study
CC1-2D	*MAT***a** *btn2::KanMX*	This study
CC10-18B	*MAT***a** *rad24::TRP1*	This study
CC10-18C	*MAT***a** *rad24::TRP1 cmr1::KanMX*	This study
CC17-5C	*MAT***a** *csm3::KanMX*	This study
CC17-9D	*MAT***a** *csm3::KanMX cmr1::NatMX*	This study
CC18-13B	*MAT***a** *elg1::KanMX*	This study
CC18-3A	*MAT***a** *elg1::KanMX cmr1::NatMX*	This study
CC2-6B	*MAT***a** *hsp42::KanMX MRC1-4ala-YFP*	This study
CC2-2B	*MAT***a** *hsp42::KanMX*	This study
CC20-13A	*MAT***a** *tof1::KanMX*	This study
CC20-13C	*MAT***a** *tof1::KanMX cmr1::NatMX*	This study
CC23-2A	*MAT***a** *dcc1::KanMX*	This study
CC23-2C	*MAT***a** *dcc1::KanMX cmr1::NatMX*	This study
CC25-1A	*MAT***a** *rad5::URA3 cmr1::KanMX*	This study
CC25-4A	*MAT***a** *rad5::URA3*	This study
CC26-11B	*MAT***a** *btn2::KanMX*	This study
CC26-2A	*MAT***a** *btn2::KanMX cmr1::KanMX*	This study
CC27-4C	*MAT***a** *cmr1::KanMX hsp42::KanMX ctf18::NatMX*	This study
CC34-3B	*MAT***a** *hel2::KanMX cmr1::NatMX*	This study
CC35-10A	*MAT***a** *ubc4::KanMX cmr1::NatMX*	This study
CC35-3D	*MAT***a** *ubc4::KanMX*	This study
CC37-5A	*MAT***a** *dia2::NatMX MRC1-4ala-YFP*	This study
CC39-3A	*MAT***a** *hel2::KanMX*	This study
CC4-19D	*MAT***a** *dia2::NatMX*	This study
CC4-3B	*MAT***a** *dia2::NatMX cmr1::KanMX*	This study
CC41-14D	*MAT***a** *mrc1AQ::LEU2::mrc1::URA3 cmr1::KanMX*	This study
CC41-6B	*MAT***a** *mrc1AQ::LEU2::mrc1::URA3*	This study
CC42-12B	*MAT***a** *rad9::HIS3*	This study
CC44-8A	*MAT***a**	This study
CC8-6D	*MAT***a** *hta1-S129A hta2-S129A cmr1::NatMX*	This study
CC98	*MAT**a** MRC1-mCherry-sfGFP::hphNT1*	This study
CC102-9C	*MAT**a** MRC1-mCherry-sfGFP::hphNT1 btn2::KanMX*	This study
DP1	*MAT***a** *cmr1::KanMX*	This study
DLY1296	*MAT***a** *ade2-1 exo1::LEU2 cdc13-1*	Ref. [5]
IG105	*MATα can1Δ::STE2pr-Sp_his5 lyp1Δ his3Δ1 leu2Δ0 ura3Δ0 met15Δ0 cmr1::NatMX*	This study**

IG106-1C	*MAT***a** *ade2-1 CAN1 lys2*$_{14A}$ *cmr1::KanMX*	This study
IG106-1D	*MAT***a** *ade2-1 CAN1 lys2*$_{14A}$ *cmr1::KanMX mlh1::NatMX*	This study
IG106-4D	*MAT***a** *ade2-1 CAN1 lys2*$_{14A}$	This study
IG106-5A	*MAT***a** *ade2-1 CAN1 lys2*$_{14A}$ *mlh1::NatMX*	This study
IG111	*MATα lys2Δ TRP1 CMR1-yEmRFP*	This study
IG118-17C	*MAT***a** *msh2::KanMX cmr1::NatMX*	This study
IG118-6C	*MAT***a** *msh2::KanMX*	This study
IG119-4D	*MAT***a** *msh6::KanMX*	This study
IG119-8D	*MAT***a** *msh6::KanMX cmr1::NatMX*	This study
IG120-2B	*MAT***a** *pms1::KanMX cmr1::NatMX*	This study
IG120-6B	*MAT***a** *pms1::KanMX*	This study
IG122-1B	*MAT***a** *msh3::KanMX*	This study
IG122-2A	*MAT***a** *msh3::KanMX cmr1::NatMX*	This study
IG137-28C	*MAT***a** *ade2-1 CAN1 lys2*$_{14A}$ *msh2::KanMX cmr1::KanMX*	This study
IG137-66D	*MAT***a** *ade2-1 CAN1 lys2*$_{14A}$ *msh2::KanMX*	This study
IG139	*MAT***a** *exo1::NatMX*	This study
IG147	*MAT***a** *MRC1-4ala-YFP*	This study
IG148	*MATα lys2Δ TRP1 MRC1-4ala-CFP*	This study
IG152-4A	*MAT***a** *exo1::NatMX cmr1::KanMX*	This study
IG154-7D	*MAT***a** *MRC1-4ala-CFP cmr1::KanMX*	This study
IG155-3A	*MAT***a** *CMR1-4ala-YFP mrc1::NatMX*	This study
IG156-6C	*MAT***a** *mrc1::NatMX cmr1::KanMX*	This study
IG156-7D	*MAT***a** *mrc1::NatMX*	This study
IG156-9B	*MATα mrc1::NatMX cmr1::KanMX*	This study
IG156-9C	*MAT***a** *lys2Δ TRP1 mrc1::NatMX cmr1::KanMX*	This study
IG160-4A	*MAT***a** *CMR1-6ala-3xYFP MRC1-4Ala-CFP*	This study
IG162-1D	*MAT***a** *rad52::his5*	This study
IG162-2D	*MAT***a** *rad52::his5 cmr1::KanMX*	This study
IG164-1D	*MAT***a** *mre11::HIS3*	This study
IG164-2B	*MAT***a** *mre11::HIS3 cmr1::KanMX*	This study
IG165-4C	*MAT***a** *rad59::HIS3*	This study
IG165-8B	*MAT***a** *rad59::HIS3 cmr1::KanMX*	This study
IG172-4B	*MAT***a** *ade2-1 CAN1 lys2*$_{14A}$ *mrc1::NatMX cmr1::KanMX*	This study
IG172-7C	*MAT***a** *ade2-1 CAN1 lys2*$_{14A}$ *mrc1::NatMX*	This study
IG174	*MAT***a** *his3Δ0 leu2Δ0 met15Δ0 ura3Δ0 Cmr1-TAP::HIS3*	Ref. [6]**
IG177-8C	*MAT***a** *ctf18::NatMX cmr1::KanMX*	This study
IG177-9C	*MAT***a** *ctf18::NatMX*	This study
IG179-3B	*MATα lys2Δ TRP1 mrc1::NatMX*	This study
IG184-11C	*MATα lys2Δ TRP1 rad52::his5 cmr1::KanMX*	This study
IG185-4C	*MAT***a** *hdf1Δ cmr1::KanMX*	This study
IG185-7C	*MAT***a** *hdf1Δ*	This study

IG186-10B	*MAT***a** *rad27::URA3 cmr1::KanMX*	This study
IG186-5D	*MAT***a** *rad27::URA3 lys2Δ*	This study
IG187-6A	*MAT***a** *rad10::KanMX*	This study
IG187-7D	*MAT***a** *rad10::KanMX cmr1::NatMX*	This study
IG188	*MAT***a** *Cmr1-YFP::NatMX*	This study
IG189-10B	*MATα can1Δ::STE2pr-LEU2 lyp1Δ his3Δ1 leu2Δ0 ura3Δ0 MET15 NLS-yEmRFPrv::HIS3 ADE2 trp1-1 CMR1-YFP::NatMX*	This study*
IG190-1C	*MAT***a** *rev3::HIS3*	This study
IG190-4D	*MAT***a** *rev3::HIS3 cmr1::KanMX*	This study
IG194-1	*MAT***a** *rad14::KanMX*	This study
IG194-2	*MAT***a** *rad14::KanMX cmr1::NatMX*	This study
IG199-1	*MAT***a** *apn1::KanMX cmr1::NatMX*	This study
IG199-2	*MAT***a** *apn1::KanMX*	This study
IG237-17C	*MAT***a** *hsp42::KanMX cmr1::KanMX*	This study
IG237-18A	*MAT***a** *hsp42::KanMX*	This study
IG238-9D	*MAT***a** *hsp42::KanMX CMR1-4ala-YFP*	This study
IG239-2B	*MAT***a** *btn2::KanMX CMR1-4ala-YFP*	This study
IG241	*MATα can1Δ::STE2pr-LEU2 lyp1Δ his3Δ1 leu2Δ0 ura3Δ0 MET15 NLS-yEmRFP::HIS3 ADE2 trp1-1 LYS2 CMR1-VC::KanMX*	This study*
IG246-2C	*MAT***a** *ubc9::TRP1 leu2-3,112-ubc9-1::LEU2 CMR1-4ala-YFP*	This study
IG250-2C	*MAT***a** *shu1::HIS3*	This study
IG250-9B	*MAT***a** *shu1::HIS3 cmr1::KanMX*	This study
IG251-3C	*MAT***a** *srs2::HIS3 cmr1::KanMX*	This study
IG253-2D	*MAT***a** *mms21-11::LEU2 cmr1::KanMX*	This study
IG256-5D	*MAT***a** *slx8::KanMX cmr1::NatMX*	This study
IG257-2C	*MAT***a** *pph3::KanMX cmr1::NatMX*	This study
IG257-9C	*MAT***a** *pph3::KanMX*	This study
IG260-1D	*MAT***a** *mms22::KanMX cmr1::NatMX*	This study
IG260-2D	*MAT***a** *mms22::KanMX*	This study
IG265-17A	*MAT***a** *rad18::KanMX*	This study
IG265-4D	*MAT***a** *rad18::KanMX cmr1::NatMX*	This study
IG266-18D	*MATα LYS2 trp1-1 ubp10::hphMX cmr1::NatMX*	This study
IG266-21A	*MATα LYS2 trp1-1 ubp10::hphMX*	This study
IG267-20D	*MAT***a** *mms2::HIS3 TRP1*	This study
IG267-9A	*MAT***a** *mms2::HIS3 cmr1::NatMX TRP1*	This study
IG285-7A	*MAT***a** *TRP1 scc1-73 cmr1::KanMX ade2-1*	This study
IG285-9A	*MAT***a** *TRP1 scc1-73 ade2-1*	This study
IG292-11B	*MAT***a** *ctf18::NatMX hsp42::KanMX*	This study
IG296-2C	*MAT***a** *pph3::KanMX dia2::NatMX*	This study
IG296-49B	*MAT***a** *pph3::KanMX dia2::NatMX cmr1::KanMX*	This study
IG299-9B	*MAT***a** *rad9::HIS3 cmr1::KanMX*	This study
IG302-1D	*MAT***a** *CMR1-4ala-CFP SLX8-4ala-YFP*	This study

IG308-12D	*MAT***a** *TRP1 mlp1::HIS3 mlp2::HIS3 cmr1::KanMX*	This study
IG308-13A	*MAT***a** *TRP1 mlp1::HIS3 mlp2::HIS3*	This study
IG310-15D	*MAT***a** *ura3::3xURA3-tetOx112 I-SceI-cs TetR-RFP cmr1::KanMX*	This study
IG309	*MAT***a** *Cmr1-7myc::KanMX*	This study
IG310-20B	*MAT***a** *ura3::3xURA3-tetOx112 I-SceI-cs TetR-RFP ctf18::NatMX*	This study
IG310-3B	*MAT***a** *ura3::3xURA3-tetOx112 I-SceI-cs TetR-RFP*	This study
IG311-1B	*MAT***a** *ura3::3xURA3-tetOx112 I-SceI-cs TetR-RFP cmr1::KanMX ctf18::NatMX*	This study
IG315	*MAT***a** *ade2-1 mrc1AQ-4ala-YFP*	This study
IG322-12A	*MAT***a** *pph3::KanMX dia2::NatMX hsp42::KanMX*	This study
IG323-19D	*MAT***a** *pph3::KanMX dia2::NatMX btn2::KanMX*	This study
IG328-4C	*MAT***a** *TRP1 Rpn11-GFP Cmr1-yEmRFP*	This study*
IG45-8A	*MAT***a** *lys2Δ*	This study
IG46-1B	*MAT***a** *lys2Δ RFA1-8ala-YFP*	This study
IG54-11D	*MAT***a** *lys2Δ RAD52-YFP*	This study
IG66	*MAT***a** *CMR1-4ala-YFP*	This study
IG71-2B	*MAT***a** *lys2Δ CMR1-YFP*	This study
IG72-5C	*MATα lys2Δ TRP1 BAR1::LEU2 RAD52-yEmRFP*	This study
IG79-6D	*MATα lys2Δ TRP1 cmr1::KanMX*	This study
IG8-5D	*MAT***a** *srs2::HIS3*	This study
LBy41	*MAT***a** *ade2-1 POL2-13myc::KanMX*	L. Bjergbæk
ML41-4B	*MAT***a** *Mec1-YFPint*	Ref. [7]
ML659-4B	*MATα can1Δ::STE2pr-LEU2 lyp1Δ his3Δ1 leu2Δ0 ura3Δ0 LYS2 MET15 NLS-yEmRFPrv::HIS3 ADE2 trp1*	This study*
ML702-R	*MAT***a** *CMR1-4ala-YFP yEmRFP-SMT3*	This study
ML8-9A	*MAT***a**	Ref. [8]
ML657	*MAT***a** *NLS-yEmRFP::HIS3*	This study
ML801-5B	*MAT***a** *Mec1-YFPint cmr1::KanMX*	This study
ML807-2D	*MAT***a** *ade2-1 cdc13-1 rpd3::KanMX*	This study
ML808-12D	*MAT***a** *ade2-1 cdc13-1 cmr1::KanMX*	This study
ML815-8A	*MAT***a** *ade2-1 cdc13-1*	This study
ML821-4C	*MAT***a** *ade2-1 cdc13-1 btn2::KanMX*	This study
ML822-4C	*MAT***a** *ade2-1 cdc13-1 hsp42::KanMX*	This study
NEB144-4C	*MAT***a** *mms21-11::LEU2*	This study
NEB248-5A	*MAT***a** *hta1-S129A hta2-S129A RAD52-YFP*	This study
NEB290-1B	*MAT***a** *slx8::KanMX*	This study
R113	*MAT***a** *cyc3-3 his1*	R. Rothstein[§]
SG936	*MATα can1Δ::STE2pr-SpHIS5 lyp1Δ ura3Δ0 leu2Δ0 his3Δ1 met15Δ0*	Steen Holmberg

SMG259-3C	*MATα lys2Δ TRP1 rad52::his5*	This study
SMG59-11B	*MAT***a** *rad52::his5*	This study
W4700-10C	*MATα lys2Δ TRP1*	Ref. [8]

* W303/S288C mixed background
** S288C background
§ W303-related background

Supplementary Table 6 Plasmids used in this study

Plasmid	Genotype	Source
Yeast plasmids		
p4339	*KAN^R AMP^R NAT^R*	Ref. [9]
pEH333	*AMP^R LEU2 hMLH1*	Ref. [1]
pEH334	*AMP^R LEU2*	Ref. [1]
pESC-VHL-GFP	*AMP^R LEU2 p-ESC-VHL-GFP*	Ref. [10]
pESC-mCherry-VHL	*AMP^R URA3 p-ESC-mCherry-VHL*	Ref. [10]
pFA6a	*AMP^R KanMX6*	Ref. [11]
pGAD-C2	*AMP^R LEU2 2μ*	Ref. [12]
pIG13	*AMP^R URA3 2μ ORI Cmr1-YFP*	This study
pIG14	*AMP^R URA3 2μ ORI $Cmr1_{NTD(1-173)}$-YFP*	This study
pIG15	*AMP^R URA3 2μ ORI $Cmr1_{SV40-NLS-WD40(174-522)}$-YFP*	This study
pIG20	*KAN^R LEU2 2μ ORI CCT6-YFP*	This study
pMaM60	*AMP^R mCherry-sfGFP-hphNT1*	Ref. [13]

pML122	KAN^R *LEU2 yEmRFP-SMT3*	This study
pML133	AMP^R *URA3 yEmRFP-SMT3*	This study
pML84	AMP^R *LEU2 ADH1p-NLS-yEmRFP 2µ*	This study
pML97	AMP^R *HIS3 2µ 3xYFP-5'-K.l. URA3*	This study
pML98	AMP^R *HIS3 2µ 3xYFP-3'-K.l. URA3*	This study
pNEB21	AMP^R *TRP1 CFP-NUP49*	Ref. [14]
pNEB30	AMP^R *HIS3 2µ yEmRFP-5'-K.l. URA3*	Ref. [7]
pNEB31	AMP^R *HIS3 2µ yEmRFP-3'-K.l. URA3*	Ref. [7]
pNEB32	AMP^R *TRP1 yEmRFP-NUP49*	This study
pRS313-3myc-Smt3	AMP^R *HIS3 2µ* myc_3*-Smt3*	Ref. [15]
pRS416	AMP^R *URA3*	Ref. [16]
pRS426	AMP^R *URA3 2µ*	Ref. [16]
pWJ1164	AMP^R *HIS3 2µ YFP-5'-K.l. URA3*	Ref. [17]
pWJ1165	AMP^R *HIS3 2µ YFP-3'-K.l. URA3*	Ref. [17]
pWJ1213	AMP^R *HIS3 Rad52-YFP*	Ref. [18]
Human plasmid		
pcDNA-DEST53-GFP-WDR76	Kan^R/Neo^R *GFP-WDR76*	This study

Supplementary Table 7 Primers used in this study

Primer name	Sequence (5' to 3')
Cmr1-VC155-fw	ATATTCCTCTTTACTGATGACTCCGGAACCATAAAGCAGGAAGAAGGTCGACGGATCCCCGGGTT
Cmr1-VC155-rv	AAAAGCGGGAGAGAAAAAGGCAGTGCGGGTAACTGAGATGTTTTCGATGAATTCGAGCTCGTT
Cmr1-YFP::NatMX-fw	GCAGAGAAAAAGCTATTTATTGTTTCTGTCCTTCGCTTTAAAACATAGAGACATGGAGGCCCAGAATACC
Cmr1-YFP::NatMX-rv	ACTGTGAGTTATGATGCATTTATAATAAGCAAT

	GATAAAAAGGACAAAATACAGTATAGCGACCAGCATTC
Cmr1up-F_HindIII	GAGAAAGCTTGGTGGCACACAATGAC
Cmr1down-R_XhoI	GAGACTCGAGCGAAGGACAGAAACAAT
Cmr1N-term-rv	ATATAGATCCAAATCAAATTC
Cmr1N-term-fw	GAATTTGATTTGGATCTATATGCTGCAGCTGCAATGAGT
Cmr1WD40-rv	ACTTTTCTTTTTTTTTTGGCATTCTTGACTGAAAAGCAGCTTTC
Cmr1WD40-fw	ATGCCAAAAAAAAAAAGAAAAGTTGAAGATCCAGACGTTTTTCAACCGAACG
CCT6_1-fw	TTGCTTAGACAAGCGCACC
CCT6_2-rv	GGTCAGTGTTCTCTGTAAC
CCT6_3-fw	TAGGCGAGGAAAAATTCACATACGTTACAGAGAACACTGACCCCGGGGGGATGAGTAAAGGAGAAGAAC
CCT6_4-rv	AGGGCATAATGAGTGGAGCCCTTGATTAAGATGGTGCAAGACTTCCCCCCTTTGTATAGTTCATCCATGC
CCT6_5-fw	AAGTCTTGCACCATCTTAATC
CCT6_6-rv	CGAATCCTGAGTTCTTCACC
NLSyEmRFP-F	TGGGTACCGCCCCAAAAAAAAAAAGAAAAGTTGAAGATCCAATGGTTTCAAAAGGTGAAGAAG
NLSyEmRFP-R	GCTAGAATTCTTATTTATATAATTCATCCATACCACC
Cmr1-13mycFW	AATATTCCTCTTTACTGATGACTCCGGAACCATAAAGCAGGAAGAACGGATCCCCGGGTTAATTAA
Cmr1-13mycRV	CTGAAAAGCGGGAGAGAAAAAAGGCAGTGCGGGTAACTGAGATGTTTGAATTCGAGCTCGTTTAAAC
Smt3-upF	CGTAGTCCCCAAGGAATAATG
SMT3-R	ATACGTAGCACCACCAATCTG
yEmRFP-Smt3-up-R	CTTCTTCACCTTTTGAAACCATCGCTCGTGTATTTATTTGTAAAAC
yEmRFP-Smt3start-F	GGTGGTATGGATGAATTATATAAAGGAGGTCCA

	GGTGGAATGTCGGACTCAGAAGTC
6ala-F-ClaI	GGTATCGATGCTGCAGCTGCAGCTGCAATG
XFP-F-EcoRI	AGGTGAATTCATGAGTAAAGGAGAAGAAC
XFP-R-XmaI	AGCCCGGGTTTGTATAGTTCATCCATGC
XFP-F-SacI	GGTGAGCTCATGAGTAAAGGAGAAGAAC
XFP-R-StuI-SphI-SacI	TAGGAGCTCTCGACGGCATGCTGCAGGCCTTTT GTATAGTTCATCCATGC
XFP-F-StuI	AGGTAGGCCTATGAGTAAAGGAGAAGAAC
XFPstop-R-SphI	AGGCATGCCTATTTGTATAGTTCATCCATGC
Cmr1-F	CCTTTCTGCGAAGTTGGAG
Cmr1-6ala-up	TGCAGCTGCAGCTGCAGCTTCTTCCTGCTTTATG GTTCC
Cmr1-3xdown	GGCATGCCGTCGAGAGCTAAACATCTCAGTTAC CCGCAC
Cmr1down-R	ACATTTTCATCCTCGGGG
6ala-F	CTGCAGCTGCAGCTGCAATG
3’-int	GAGCAATGAACCCAATAACGAAATC
5’-int	CTTGACGTTCGTTCGACTGATGAGC
term-R	AGCTCTCGACGGCATGCC
Mrc1-S3-F	TCGAATAAACTTTTTGAAAGCGGACAAGATAGC TTTGATAATCGTACGCTGCAGGTCGAC
Mrc1-S2-R	GGAGTTCAATCAACTTCTTCGGAAAAGATAAAA AACCACTAATCGATGAATTCGAGCTCG

Supplementary Methods

Sister chromatid cohesion assay

Defects in sister chromatid cohesion were analyzed[3] in strains carrying *TetO* tandem repeats on chromosome *V*, 110 kb from the centromere (*URA3* locus), and constitutively expressing TetR-RFP were grown to exponential phase, arrested in G2 with 8 µg ml^{-1} nocodazole for 2.5 hours and imaged.

Chromatin immunoprecipitation.

ChIP was performed as described before[19]. Briefly, cells were synchronized in G1 with α-factor at 30°C, washed and released in pre-warmed YPD medium containing 0.2 M HU for 10, 15, 30 and 60 minutes before fixation with 1% formaldehyde. Monoclonal anti-Myc antibody (9E10, Santa Cruz Biotechnology) was incubated with whole cell extract before coupling with equilibrated Dynabeads. Samples incubated with Dynabeads without antibody were used as background control. Real-time quantitative PCR was performed at the early-replicating ARS305, at regions 3 kb upstream and downstream ARS305 and at late-replicating ARS501, using a CFX96 Real-time System (BioRad). The average of two to four independent real-time PCR measurements and standard deviation is reported. Fold increase of the IP over the beads-only control was calculated as before[19] using the formula: fold increase = $2^{(C_T\text{input} - C_T\text{IP})} / 2^{(C_T\text{input} - C_T\text{beads})}$.

Flow cytometry

Cultures were harvested by centrifugation and fixed in 70% ethanol overnight at 4°C. Fixed cells were washed and resuspended in 50 mM NaCitrate (pH 7) prior to sonication and addition of RNase A (Sigma) to a final concentration of 250 µg ml^{-1}. Cells were incubated for 1 hour at 55°C followed by addition of Proteinase K (Sigma) to a final concentration of 1 mg ml^{-1} and further incubation at 55°C for 1 hour. Cells were either stored at 4°C or directly stained with propidium iodide (PI, Sigma) by adding 20 µl cell suspension to 500 µl 50 mM NaCitrate (pH 7) containing 16 µg ml^{-1} PI. Sample PI profiles were recorded using a FACSCalibur (BD Biosciences). Data were processed using FlowJo software (10.0.6).

Supplementary References:

1. Shimodaira, H. *et al.* Functional analysis of human MLH1 mutations in Saccharomyces cerevisiae. *Nat Genet* **19**, 384-389 (1998).
2. Maere, S., Heymans, K. & Kuiper, M. BiNGO: a Cytoscape plugin to assess overrepresentation of gene ontology categories in biological networks. *Bioinformatics (Oxford, England)* **21**, 3448-3449 (2005).
3. Parnas, O. *et al.* The ELG1 clamp loader plays a role in sister chromatid cohesion. *PLoS One* **4**, e5497 (2009).
4. Thomas, B.J. & Rothstein, R. Elevated recombination rates in transcriptionally active DNA. *Cell* **56**, 619-630 (1989).
5. Zubko, M.K., Guillard, S. & Lydall, D. Exo1 and Rad24 differentially regulate generation of ssDNA at telomeres of *Saccharomyces cerevisiae cdc13-1* mutants. *Genetics* **168**, 103-115 (2004).
6. Ghaemmaghami, S. *et al.* Global analysis of protein expression in yeast. *Nature* **425**, 737-741 (2003).
7. Silva, S., Gallina, I., Eckert-Boulet, N. & Lisby, M. Live Cell Microscopy of DNA Damage Response in *Saccharomyces cerevisiae*. *Methods Mol Biol* **920**, 433-443 (2012).
8. Germann, S.M. *et al.* Dpb11/TopBP1 plays distinct roles in DNA replication, checkpoint response and homologous recombination. *DNA Repair (Amst)* **10**, 210-224 (2011).
9. Tong, A.H. *et al.* Systematic genetic analysis with ordered arrays of yeast deletion mutants. *Science* **294**, 2364-2368. (2001).
10. Kaganovich, D., Kopito, R. & Frydman, J. Misfolded proteins partition between two distinct quality control compartments. *Nature (London)* **454**, 1088-1095 (2008).
11. Bahler, J. *et al.* Heterologous modules for efficient and versatile PCR-based gene targeting in *Schizosaccharomyces pombe*. *Yeast* **14**, 943-951 (1998).
12. James, P., Halladay, J. & Craig, E.A. Genomic libraries and a host strain designed for highly efficient two-hybrid selection in yeast. *Genetics* **144**, 1425-1436 (1996).
13. Khmelinskii, A. *et al.* Tandem fluorescent protein timers for in vivo analysis of protein dynamics. *Nat Biotechnol* **30**, 708-714 (2012).
14. Khadaroo, B. *et al.* The DNA damage response at eroded telomeres and tethering to the nuclear pore complex. *Nat. Cell Biol.* **11**, 980-987 (2009).
15. Panse, V.G., Hardeland, U., Werner, T., Kuster, B. & Hurt, E. A proteome-wide approach identifies sumoylated substrate proteins in yeast. *J Biol Chem* **279**, 41346-41351 (2004).
16. Sikorski, R.S. & Hieter, P. A system of shuttle vectors and yeast host strains designed for efficient manipulation of DNA in *Saccharomyces cerevisiae*. *Genetics* **122**, 19-27 (1989).
17. Lisby, M., Rothstein, R. & Mortensen, U.H. Rad52 forms DNA repair and recombination centers during S phase. *Proc. Natl. Acad. Sci. USA* **98**, 8276-8282 (2001).
18. Feng, Q. *et al.* Rad52 and Rad59 exhibit both overlapping and distinct functions. *DNA Repair (Amst)* **6**, 27-37 (2007).

19. Cobb, J.A., Bjergbaek, L., Shimada, K., Frei, C. & Gasser, S.M. DNA polymerase stabilization at stalled replication forks requires Mec1 and the RecQ helicase Sgs1. *EMBO J.* **22**, 4325-4336 (2003).
